# Lactate programs CRIP1 protein lactylation to drive synovial proliferation in rheumatoid arthritis

**DOI:** 10.1172/jci.insight.200928

**Published:** 2026-06-08

**Authors:** Meican Ma, Yu Zhou, Qianlin Li, Zhao Wang, Shangqi Guan, Xiaoxue Wang, Han Zhao, Zhenke Wen, Ting Liu, Fenghong Yuan

**Affiliations:** 1 Department of Rheumatology and Immunology and; 2 Department of Clinical Research Center, The Affiliated Wuxi People’s Hospital of Nanjing Medical University, Wuxi People’s Hospital, Wuxi Medical Center, Nanjing Medical University, Wuxi, Jiangsu, China.; 3 Department of Rheumatology and Immunology, National Clinical Research Center for Infectious Disease, Shenzhen Third People’s Hospital, Shenzhen, Guangdong, China.; 4 Department of Rheumatology and Immunology, The Second Affiliated Hospital of Harbin Medical University, Harbin Medical University, Harbin, Heilongjiang, China.; 5 Department of Endocrinology and Metabolism, The Affiliated Wuxi People’s Hospital of Nanjing Medical University, Wuxi People’s Hospital, Wuxi Medical Center, Nanjing Medical University, Wuxi, Jiangsu, China.; 6 The Fourth Affiliated Hospital of Soochow University, Institutes of Biology and Medical Sciences, Suzhou Medical College of Soochow University, Soochow University, Suzhou, Jiangsu, China.; 7 Jiangsu Key Laboratory of Infection and Immunity, MOE Key Laboratory of Geriatric Diseases and Immunology, Biomedical Basic Research Center of Jiangsu Province, Soochow University, Suzhou, Jiangsu, China.

**Keywords:** Autoimmunity, Metabolism, Rheumatology

## Abstract

Synovial hyperplasia is a hallmark of rheumatoid arthritis (RA), yet its mechanism remains unclear. RA synovium exhibits metabolic shift, characterized by upregulated glycolysis and enhanced lactate production. In this study, we elucidated the mechanism underlying the roles of lactate metabolism and protein lactylation in RA pathology. In patients with RA, both lactate production and protein lactylation were elevated and showed a positive correlation with clinical disease activity. These changes were further implicated in driving synovial proliferation. Among the lactylated proteins, Cysteine-rich intestinal protein 1 (CRIP1) exhibited a marked increase in modification and played a central role in promoting synovial proliferation. Mechanistically, CRIP1 underwent MOF-mediated lactylation in RA synovial fibroblasts. Lactylated CRIP1 hijacked the cell-cycle regulator p21, disrupting its interaction with cyclin-dependent kinase 2 (CDK2), thereby facilitating the G1/S phase transition. Functionally, AAV-mediated delivery of a lactylation-deficient CRIP1 K49R significantly reduced synovial proliferation compared with WT CRIP1. Peptide-based interventions targeting CRIP1 K49 lactylation effectively inhibited synovial hyperplasia and disease severity in both Collagen II–induced arthritis (CIA) and humanized NSG chimeric models. Collectively, CRIP1 protein lactylation drives synovial proliferation in RA by hijacking p21 from CDK2, thereby facilitating cell cycle progression. Targeting this pathway may serve as a promising strategy for RA.

## Abstract 2

In rheumatoid arthritis, CRIP1 lactylation drives synovial proliferation by diverting p21 from CDK2 to advance the cell cycle—a promising therapeutic target.

## Graphical abstract

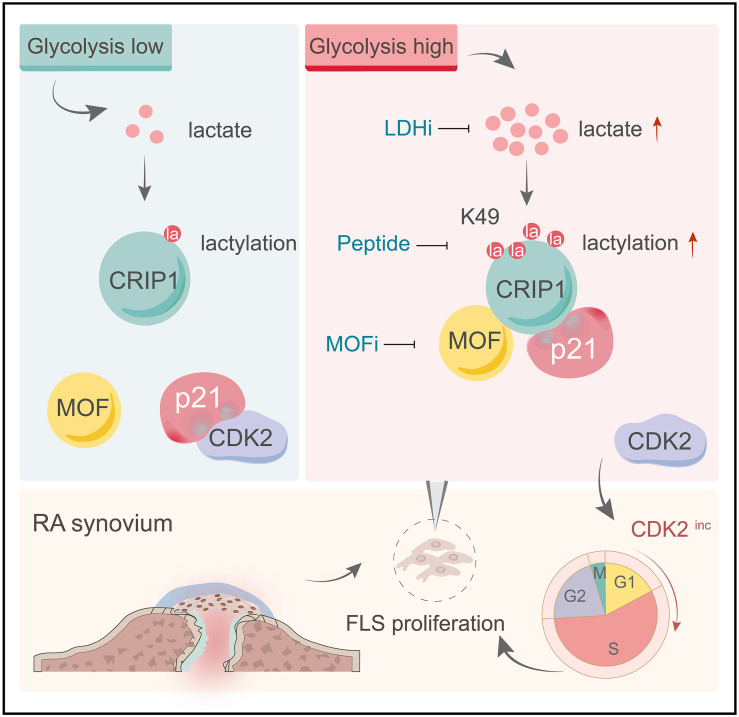

## Introduction

Rheumatoid arthritis (RA) is a systemic autoimmune disease primarily affecting the joints, resulting in progressive destruction and functional impairment (1, 2). RA imposes a substantial burden on individuals, society, and healthcare system (3). Current therapies, predominantly conventional synthetic disease-modifying antirheumatic drugs (csDMARDs) and biologic/targeted synthetic DMARDs (b/tsDMARDs), aim to modulate immune responses and inflammatory signaling (4). However, up to 30%–40% of patients fail to achieve satisfactory clinical outcomes (5). Nonresponders frequently present with a pauci-immune phenotype, characterized by persistent synovial proliferation (6). This suggests that targeting the local environment, particularly the resident stromal cells, represents a promising therapeutic approach.

Synovial fibroblasts (fibroblast-like synoviocytes [FLSs]) play a crucial role in the development of RA (7). RA-FLSs are characterized by several pathological features, including enhanced proliferation, invasion, inflammatory activation, and resistance to apoptosis (8). These cells undergo transformation, acquiring cancer-like properties driven by epigenetic modifications, metabolic reprogramming, and complex interactions with both immune cells and neighboring FLSs, which actively promote synovial hyperplasia and articular tissue degradation (8). Extensive FLS expansion leads to the formation of pannus, a hypertrophied synovial tissue that attaches to and invades cartilage and bone (9). Additionally, FLSs secrete a range of destructive mediators, including matrix metalloproteinases (MMP-1, MMP-9, MMP-13), aggrecanases (ADAMTS-4, ADAMTS-5), and receptor activator of nuclear factor κB ligand (RANKL), all of which contribute directly to irreversible joint damage in RA (10). Despite these insights, no FLS-directed therapies have been approved (11). Elucidating the specific pathways and factors governing synovial proliferation in RA may facilitate the development of more precise and effective treatments for autoimmune joint disorders.

In RA, FLSs undergo distinct metabolic rewiring, a shift toward an anabolic, activated state, thereby promoting aberrant cellular proliferation (12). Specifically, these cells manifest upregulated glycolysis flux and enhanced pentose phosphate pathway, alongside impaired mitochondrial function (13). Hypoxic conditions, nutrient deprivation, and proinflammatory factors, such as IL-6, IL-1β, TNF, IL-17, and TLRs, may contribute to these metabolic changes (14, 15). The Warburg effect, characterized by increased glycolytic enzymes and elevated levels of metabolites, including lactate, pyruvate, and acetyl-CoA, has been reported in numerous studies (16). Of note, lactate is known to function as an important signaling molecule, modulating the activities of infiltrating immune and tissue-resident cells (17). Synovial fluid from patients with RA shows elevated lactate/glucose ratios, with a reduction of pO_2_ and pH, strongly correlate with disease activity and proinflammatory cytokines (12). Monocarboxylate transporter 4 (MCT4), responsible for lactate export into the extracellular space, is upregulated in RA-FLS (18). Results from studies on the loss of MCT4 have demonstrated its involvement in synovial hyperplasia and cartilage damage (19). However, the precise mechanism linking lactate to FLS proliferation remains to be realized.

Lactylation, a recently defined posttranslational modification (PTM), has introduced new significance to metabolic reprogramming (20). Lactate-derived lactyl-CoA serves as the acyl donor for the ε-amino group of lysine lactylation, establishing a mechanistic connection between metabolism and cellular function (21–23). Accumulating evidence over recent years points to a crucial role for protein lactylation in controlling cell proliferation (23). For instance, lactylation of p53 disrupts its subcellular localization and transcriptional activity, contributing to gene expression programs associated with tumor growth (24, 25). Similarly, lactylation of the DNA repair protein MRE11 at K673, impairs cell cycle checkpoints and promotes tumor resistance (26, 27). A growing number of lactylated proteins have been identified, including those involved in chromatin remodeling, cell cycle control, and protein folding, such as heat shock proteins (HSPs) (28). Research to date has primarily focused on the role of lactylation in immune cell regulation, particularly through the modification of histones and lesser-known nonhistone proteins (22, 29). For example, Th17 cells can differentiate into Tregs through H3K18 lactylation — a process in which IL-2 signaling suppresses IL-17 production while enhancing Foxp3 expression (30). Lactate-induced lactylation also promotes the stabilization of pyruvate kinase M2 (PKM2) into its active tetrameric form in macrophages (31). Notably, lactylation of metabolic regulators such as NDUFB3, NGLY1, and SLC25A4 has been found to be highly enriched in plasma cells from patients with RA (32). Lactylation at FTH1-K69 and PKM2-K166 has been reported to be elevated in the synovium of patients with RA compared with those with osteoarthritis (OA) (33). However, the functional relevance of protein lactylation within RA synovium remains largely unexplored.

This study sought to uncover the mechanisms driving synovial proliferation in patients with RA. Elevated lactate production and enhanced protein lactylation in RA were associated with synovial proliferation. We pinpointed that lactylation of cysteine-rich intestinal protein 1 (CRIP1) mediated this proliferative phenotype by sequestering p21 from cyclin-dependent kinase 2 (CDK2), thus facilitating the G1/S phase transition in RA-FLSs. A functional cell-penetrating peptide (CPP), designed to selectively inhibit CRIP1 protein lactylation, demonstrated therapeutic efficacy in RA models. These findings shed light on the complex mechanisms in synovial proliferation and lay the groundwork for targeted therapies in patients with RA.

## Results

### Elevated lactate generation and robust protein lactylation portend synovial proliferation.

**Figure 1:**
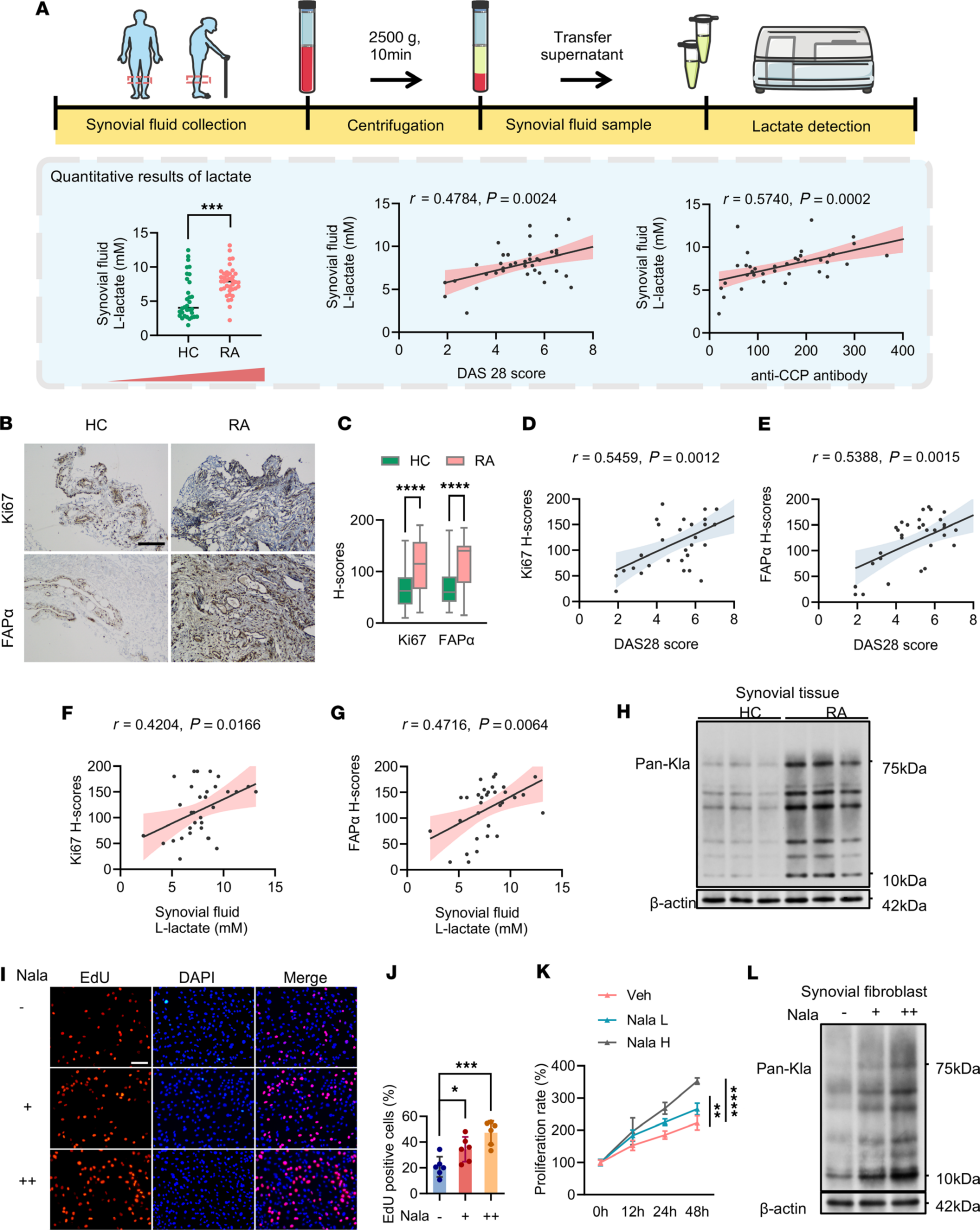
Enhanced lactate production and heightened protein lactylation drive synovial proliferation. (**A**) Quantification of synovial fluid lactate and its clinical correlation in RA. Schematic workflow illustrating synovial fluid collection, centrifugation (2,500 × *g*, 10 minutes), and lactate measurement. L-lactate levels in synovial fluid from patients with RA (*n* = 38) and healthy controls (HC, *n* = 32); left panel. Correlation between synovial lactate and DAS28 in patients with RA (*n* = 38); middle panel. Association between synovial lactate and serum anti-CCP antibody levels (*n* = 38); right panel. (**B** and **C**) IHC staining and quantification of Ki67 and FAPα in synovial tissues from patients with RA and healthy controls (*n* = 32). Scale bar: 200 μm. (**D** and **E**) Correlation of Ki67 and FAPα expression with DAS28 in patients with RA (*n* = 32). (**F** and **G**) Correlation of Ki67 and FAPα expression with synovial lactate levels in patients with RA (*n* = 32). (**H**) Immunoblot analysis of Pan-Kla levels in synovial tissues from patients with RA and healthy controls (*n* = 6). (**I** and **J**) EdU incorporation assay and quantification of proliferation in RA-FLS treated with Nala (10 mM, 20 mM) (*n* = 6). Scale bar: 100 μm. (**K**) CCK8 assay to assess cell proliferation following Nala treatment (10 mM, 20 mM) (*n* = 6). (**L**) Immunoblot analysis of Pan-Kla in RA-FLSs treated with Nala (10 mM, 20 mM) (*n* = 3). *P < 0.05, **P < 0.01, ***P < 0.001, ****P < 0.0001. Data are presented as mean ± SEM, and *P* values are calculated using unpaired 2-tailed *t* test (**A** [left] and **C**), or Pearson’s correlation (**A** [middle and right] and **D**–**G**), or 1-way ANOVA followed by Tukey’s post hoc test (**J**), 2-way ANOVA with Bonferroni’s post hoc test (**K**).

To investigate the role of metabolic changes in RA pathogenesis, we examined lactate levels in synovial fluid and found a marked elevation in patients with RA relative to healthy individuals. Such synovial lactate of patients with RA showed a positive correlation with both Disease Activity Score in 28 joints (DAS28) and anticyclic citrullinated peptide (anti-CCP) antibodies (Figure 1A). Subsequently, synovial tissues were harvested, and samples from patients with RA showed increased expression of Ki67 and FAPα (Figure 1, B and C). As anticipated, these synovial proliferation markers correlated with DAS28 (Figure 1, D and E). Of interest, synovial lactate in patients with RA were positively linked to these proliferation markers (Figure 1, F and G). Since lactate serves as a direct precursor for lactyl-CoA and subsequent protein lactylation, we next examined changes in protein lactylation levels. In line with aforementioned results, protein lactylation was markedly elevated in RA synovial tissues (Figure 1H and Supplemental Figure 1A; supplemental material available online with this article; https://doi.org/10.1172/jci.insight.200928DS1). Given the central role of FLSs in synovial hyperplasia, we next examined the functional effect of lactate on RA-FLSs. Primary RA-FLSs were isolated from synovial tissues, and intracellular lactate levels were manipulated using the lactate donor Nala. Consistent with the tissue-level observations, Nala stimulation significantly promoted RA-FLS proliferation in a dose-dependent manner (Figure 1, I–K). In contrast, we did not detect a significant effect on apoptosis of RA-FLSs (Supplemental Figure 1, B and C). In addition, Nala modulated protein lactylation in RA-FLSs in a manner that aligned with their effects on cell proliferation (Figure 1L and Supplemental Figure 1D), further supporting the role of protein lactylation in synovial hyperplasia.

### Inhibition of lactate production suppresses synovial proliferation.

**Figure 2:**
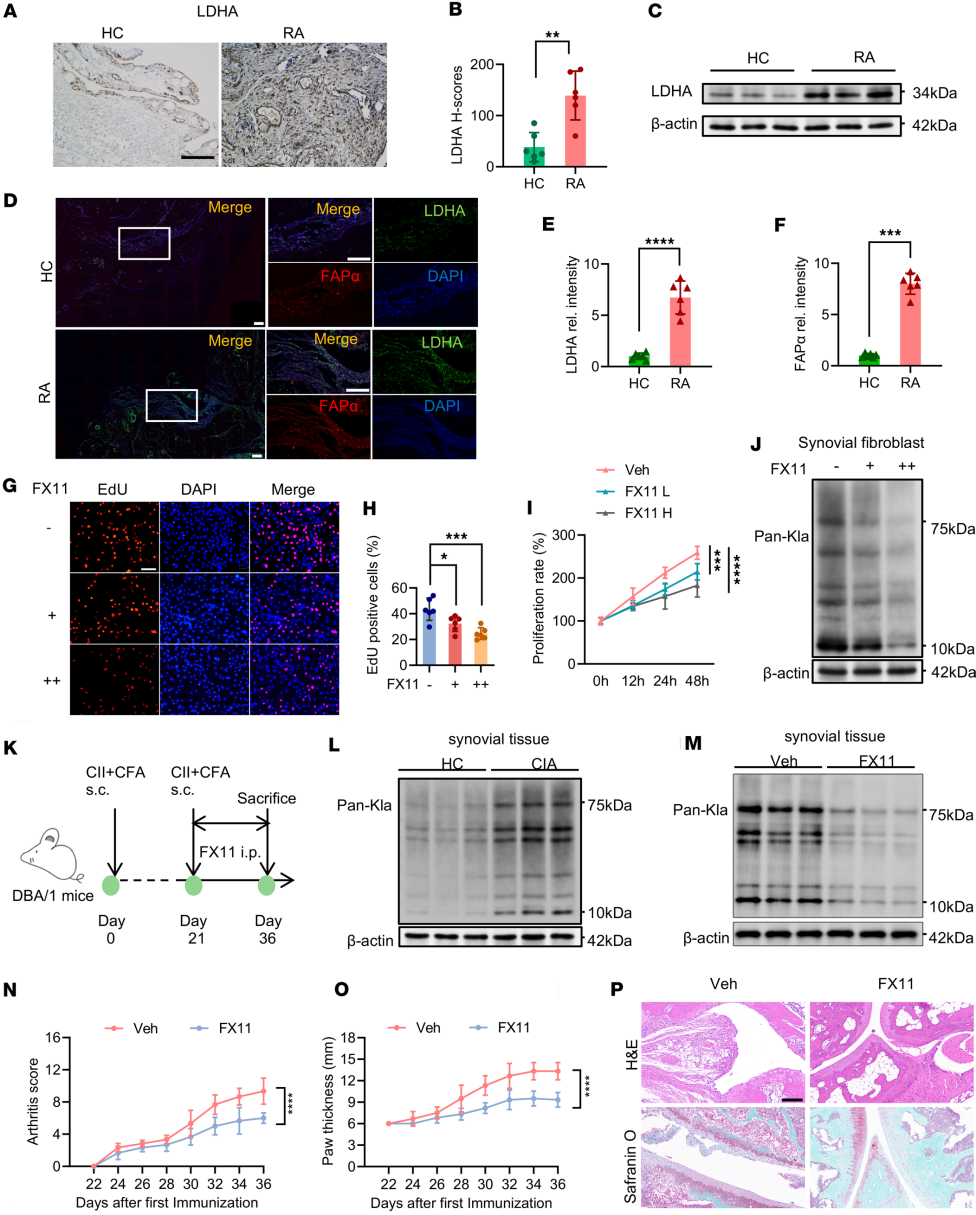
Inhibition of lactate metabolism reduces synovial proliferation. (**A** and **B**) IHC staining and quantification of LDHA in synovial tissues from patients with RA and healthy controls (*n* = 6). Scale bar: 200 μm. (**C**) Immunoblot analysis of LDHA in synovial tissues from patients with RA and healthy controls (*n* = 6). (**D**–**F**) Immunofluorescence staining and quantification of LDHA (green) and FAPα (red) in synovial tissues from patients with RA and healthy controls (*n* = 6). Scale bar: 200 μm. (**G** and **H**) EdU incorporation assay and quantification of RA-FLS treated with FX11 (10 μM, 20 μM) (*n* = 6). Scale bar: 100 μm. (**I**) CCK8 assay to assess proliferation cells treated with FX11 (10 μM, 20 μM) (*n* = 6). (**J**) Immunoblot analysis of Pan-Kla in synovial tissues upon FX11 treatment (*n* = 3). (**K**) Establishment of a CIA mouse model. Mice were immunized s.c. with type II collagen emulsified in complete Freund’s adjuvant (CII + CFA) on day 0 and again on day 21. FX11 (1 mg/kg, Qd) or vehicle was administered i.p. on day 21. Mice were sacrificed on day 36 for treatment evaluation. (**L**) Immunoblot analysis of Pan-Kla in synovial tissues from HC and CIA mice (*n* = 3). (**M**) Immunoblot analysis of Pan-Kla in synovial tissues upon FX11 treatment (*n* = 3). (**N** and **O**) Clinical arthritis score and paw thickness upon FX11 treatment (*n* = 6). (**P**) Histological staining with H&E and Safranin O in synovial tissues upon FX11 treatment (*n* = 6). Scale bar: 100 μm. *P < 0.05, **P < 0.01, ***P < 0.001, ***P < 0.001. Data are presented as mean ± SEM, and *P* values are calculated using unpaired 2-tailed *t* test (**B**, **E**, and **F**), or 1-way ANOVA followed by Tukey’s post hoc test (**H**), or 2-way ANOVA with Bonferroni’s post hoc test (**I**, **N**, and **O**).

Given that lactate dehydrogenase A (LDHA) directly catalyzes lactate production, we examined its expression in synovial tissues. LDHA levels were significantly higher in RA samples compared with healthy controls, a finding further validated at the tissue level using IHC, immunoblotting, and immunofluorescence (Figure 2, A–F, and Supplemental Figure 1E). Building on these findings, we next investigated the functional role of lactate inhibition in RA-FLSs. Treatment with the selective LDHA inhibitor FX11 significantly reduced cell proliferation in a dose-dependent manner (Figure 2, G–I), along with a parallel decrease in protein lactylation (Figure 2J and Supplemental Figure 1F). We further extended these findings in vivo using the classical CIA mouse model (Figure 2K). Protein lactylation was markedly increased in CIA mice but was declined upon FX11 administration (Figure 2, L and M, and Supplemental Figure 1, G and H). Consistently, FX11 treatment reduced arthritis severity in CIA mice, as reflected by decreased clinical scores and paw thickness (Figure 2, N and O). These effects were further validated through histological examination, bolstering the structural basis for therapeutic effects (Figure 2P). Collectively, inhibition of lactate production and vigorous protein lactylation suppresses synovial proliferation in RA.

### CRIP1 protein lactylation controls synovial proliferation in RA.

**Figure 3:**
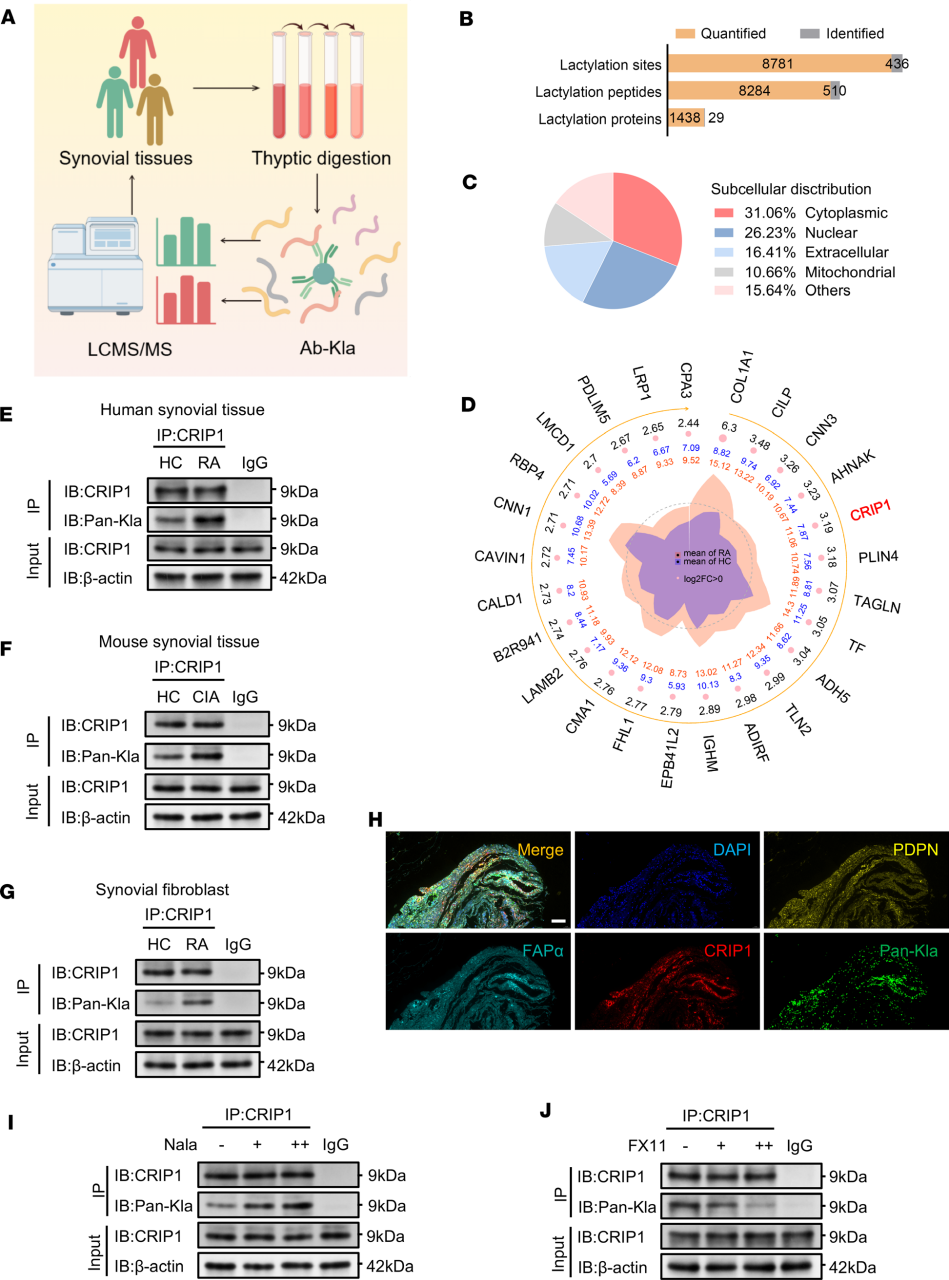
CRIP1 protein lactylation promotes synovial proliferation in RA. (**A**) Schematic workflow of lactylation proteomics in synovial tissues from patients with RA and healthy controls. (**B**) Summary of identified and quantified lactylation sites, peptides, and proteins. (**C**) Subcellular distribution of lactylated proteins. (**D**) Radar chart of 25 representative lactylated proteins with elevated modification levels in patients with RA. (**E** and **F**) Co-IP of CRIP1 followed by immunoblotting for Pan-Kla to detect CRIP1 protein lactylation in human (**E**) and mouse synovium (**F**). (**G**) Co-IP of CRIP1 followed by immunoblotting for Pan-Kla to detect CRIP1 protein lactylation in FLSs from patients with RA and healthy controls (*n* = 3). (**H**) Immunofluorescence staining of CRIP1 (red), Pan-Kla (green), PDPN (yellow), FAPα (cyan) in RA synovium. Scale bar: 100 μm. (**I**) Co-IP of CRIP1 followed by immunoblotting for Pan-Kla to detect CRIP1 lactylation in RA-FLSs after Nala treatment (10 mM, 20 mM) (*n* = 3). (**J**) Co-IP of CRIP1 followed by immunoblotting for Pan-Kla to detect CRIP1 lactylation in RA-FLSs after LDHA inhibition with FX11 (10 μM, 20 μM) (*n* = 3).

To systemically map the role of protein lactylation in RA, we conducted a comprehensive lactylome analysis on freshly obtained synovial tissue samples from both HC and patients with RA. Synovial tissues exhibiting hyperplasia were selected for this study. Proteins were digested using trypsin, and the resulting peptides were selectively enriched using antilactyl lysine antibodies. The enriched peptides were then analyzed by LC-MS/MS, which led to the identification and quantification of altered lactylation sites (Figure 3A). A total of 9,217 lysine lactylation sites were identified across 1,467 proteins. Among these, 8,781 sites in 1,438 proteins provided high-confidence quantitative data (localization probability > 0.75, FDR < 1%) (Figure 3B), which enabled robust identification of differentially lactylated proteins in subsequent analyses. Subcellular localization analysis revealed that lactylated proteins were predominantly situated in the cytoplasm (31.06%) and nucleus (26.23%), with additional representation in mitochondrial and extracellular compartments (Figure 3C). The broad subcellular distribution indicated that lactylation extensively affected nonhistone proteins involved in diverse cellular processes. Relative quantification identified the top 25 differentially expressed lactylated proteins (DELPs) that were upregulated in RA. Among them, COL1A, CILP, CNN3, AHNAK, and CRIP1 showed the most prominent increases (Figure 3D). These highly lactylated proteins were subsequently prioritized for validation and mechanistic investigation in the context of synovial proliferation.

We performed sequential genetic knockdown experiments, and transfection efficiency was validated at both the mRNA and protein levels (Supplemental Figure 2). CRIP1 silencing resulted in a discernible reduction in cell proliferation, while no such effect was observed with the other candidates (Supplemental Figure 3, A and B). Besides, CRIP1 knockdown did not significantly affect other phenotypes of RA-FLSs, including apoptosis, inflammation markers IL-6 and TNF, or invasion (Supplemental Figure 3, C–H). These findings pinpoint a CRIP1-dependent mechanism by which protein lactylation controls synovial proliferation. CRIP1 is a zinc-binding protein containing a LIM domain that mediates protein–protein interactions and is involved in various cellular processes, including tumor proliferation, metastasis, autophagy, and inhibition of apoptosis (34). To further investigate the role of CRIP1 in RA, we conducted the following experiments. Immunoblotting results unveiled elevated CRIP1 protein lactylation in synovial tissues from both patients with RA and CIA mice (Figure 3, E and F). A similar pattern was observed in RA-FLSs when compared with healthy controls (HC) and patients with OA (Figure 3G and Supplemental Figure 4, A–C). Thus, we reasonably linked CRIP1 protein lactylation with RA-FLS proliferation. Corroborating this, single-cell transcriptomic analysis revealed the predominant CRIP1 expression in FLS clusters within RA synovium (Supplemental Figure 4D). Immunofluorescence analysis visually validated the colocalization of CRIP1 and lysine lactylation in pathogenic FLSs expressing FAPα and PDPN (Figure 3H). Additionally, immunoblotting of immune cells (macrophages, T cells, and B cells) did not show an increase in CRIP1 lactylation (Supplemental Figure 4, E–G). Next, we modulated protein lactylation levels in RA-FLSs, yielding a tendency comparable with that in cell proliferation. Nala treatment dose-dependently enhanced CRIP1 protein lactylation; vice versa, FX11 led to its gradual reduction (Figure 3, I and J, and Supplemental Figure 4, H and I). Based on these observations, CRIP1 protein lactylation is the critical basis for heightened proliferation in RA-FLSs.

### CRIP1 K49 lactylation confers synovial proliferation in RA.

**Figure 4:**
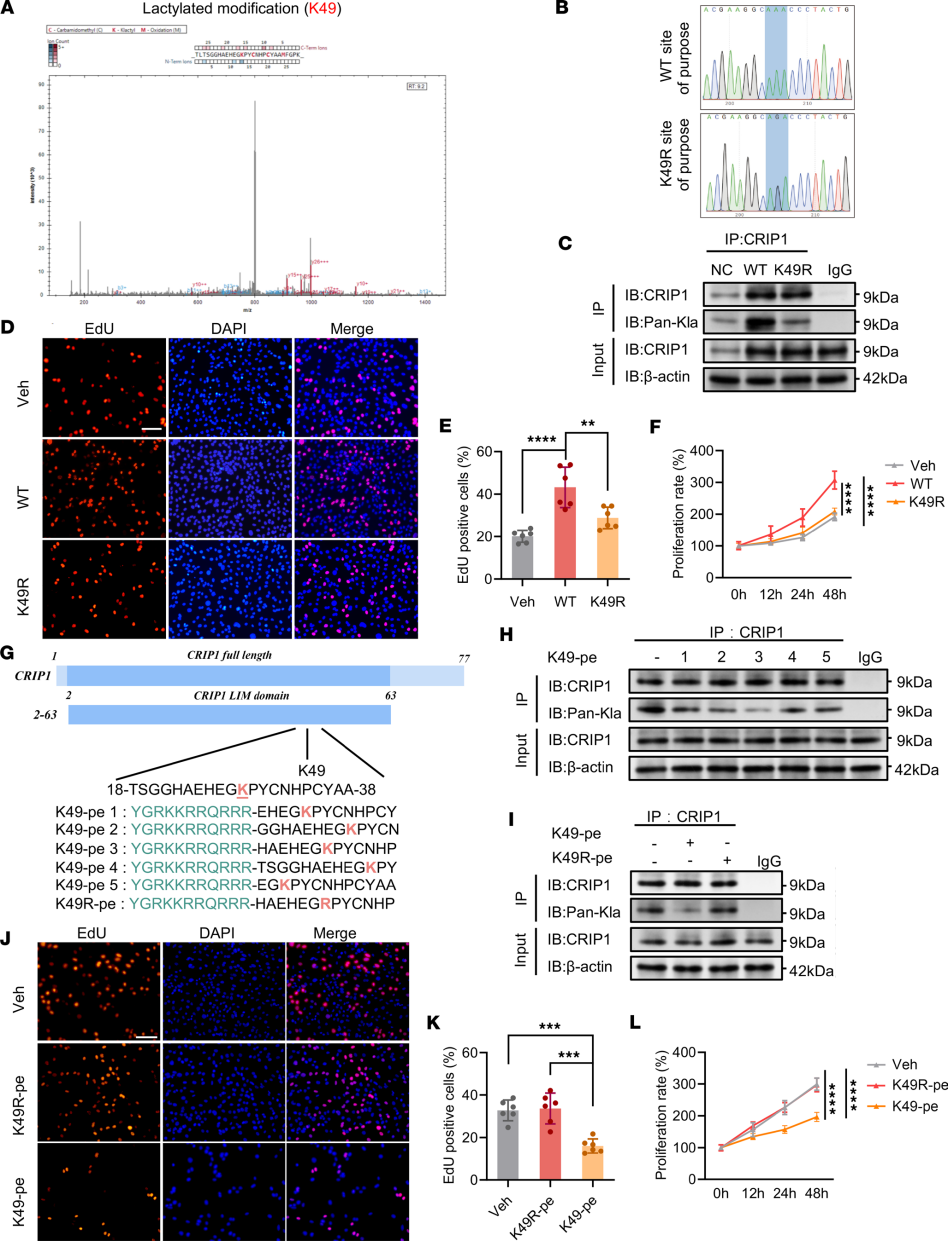
CRIP1 K49 lactylation facilitates synovial proliferation in RA. (**A**) MS/MS spectrum of lysine lactylation at the K49 locus in CRIP1. (**B**) Sanger sequencing of the AAG to AGG substitution at the K49 locus in CRIP1. (**C**) Co-IP of CRIP1 followed by immunoblotting for Pan-Kla to assess CRIP1 protein lactylation in RA-FLSs expressing WT or CRIP1 K49R. (**D** and **E**) EdU incorporation assays and quantification of cell proliferation in RA-FLSs expressing Veh, WT and CRIP1 K49R (*n* = 6). Scale bar: 100 μm. (**F**) CCK-8 assay to quantify cell proliferation in RA-FLSs expressing Veh, WT, and CRIP1 K49R (*n* = 6). (**G**) Schematic presentation of CRIP1 protein domains and sequence of the K49 region. Synthetic peptides (K49-peptide 1–5, K49R-peptide 6) were designed for the study. (**H**) Co-IP of CRIP1 followed by immunoblotting for Pan-Kla to detect CRIP1 protein lactylation upon treatment with K49-peptide 1–5. (**I**) Co-IP of CRIP1 followed by immunoblotting for Pan-Kla showing CRIP1 protein lactylation after treatment with K49R or K49-peptide. (**J** and **K**) EdU incorporation assay and quantification of cell proliferation in RA-FLSs treated with Veh, K49R, or K49-peptide (*n* = 6). Scale bar: 100 μm. (**L**) CCK-8 assay to quantify cell proliferation in RA-FLSs treated with Veh, K49R, or K49-peptide (*n* = 6). **P < 0.01, ***P < 0.001, ****P < 0.0001. Data are presented as mean ± SEM, and *P*-values are calculated using 1-way ANOVA followed by Tukey’s post hoc test (**E** and **K**), or 2-way ANOVA with Bonferroni’s post hoc test (**F** and **L**).

To understand the intricacies of lactylation mechanism, we conducted in-depth analysis and, based on lactylome profiling, identified 5 residues (K9, K22, K30, K49, K77) upregulated in CRIP1 (Figure 4A and Supplemental Figure 5, A–E). Site-directed mutagenesis (K→R) was sequentially performed to mimic a nonlactylated form in these 5 candidate residues. Among them, K49 mutant exhibited the sharpest decrease in CRIP1 protein lactylation (Supplemental Figure 5, F and G). Thus, we postulated that CRIP1 K49 lactylation leads to the aberrant activity in RA-FLSs.

Protein sequence alignment confirmed the evolutionary conservation of K49 underscoring its relevance to disease pathogenesis (Supplemental Figure 5H). Subsequently, RA-FLSs were transfected with lentiviruses expressing WT or CRIP1 K49R (Figure 4, B and C). Proliferative rate was markedly lower in the K49R group compared with the WT group (Figure 4, D–F). Based on these observations, which implicated CRIP1 K49 lactylation in the aberrant activation of RA-FLSs, we reasoned that selective disruption of this PTM might provide a means to modulate CRIP1-associated functions. Accordingly, 5 synthetic peptides around K49 site were designed to competitively interfere with CRIP1 K49 lactylation (Figure 4G and Supplemental Figure 6). By immunoblotting, CRIP1 protein lactylation was shown to be inhibited by multiple peptides, with peptide 3 being the most effective (Figure 4H). And, this peptide 3 was used in the following K49-peptide experiments. K49-peptide reduced CRIP1 protein lactylation, whereas the K49R-peptide had no effect (Figure 4I). Consistent with the lactylation results, the K49 peptide significantly suppressed cell proliferation, while the K49R peptide did not (Figure 4, J–L). In combination, CRIP1 K49 lactylation controls synovial proliferation in RA.

### MOF is essential for CRIP1 protein lactylation and synovial proliferation.

**Figure 5:**
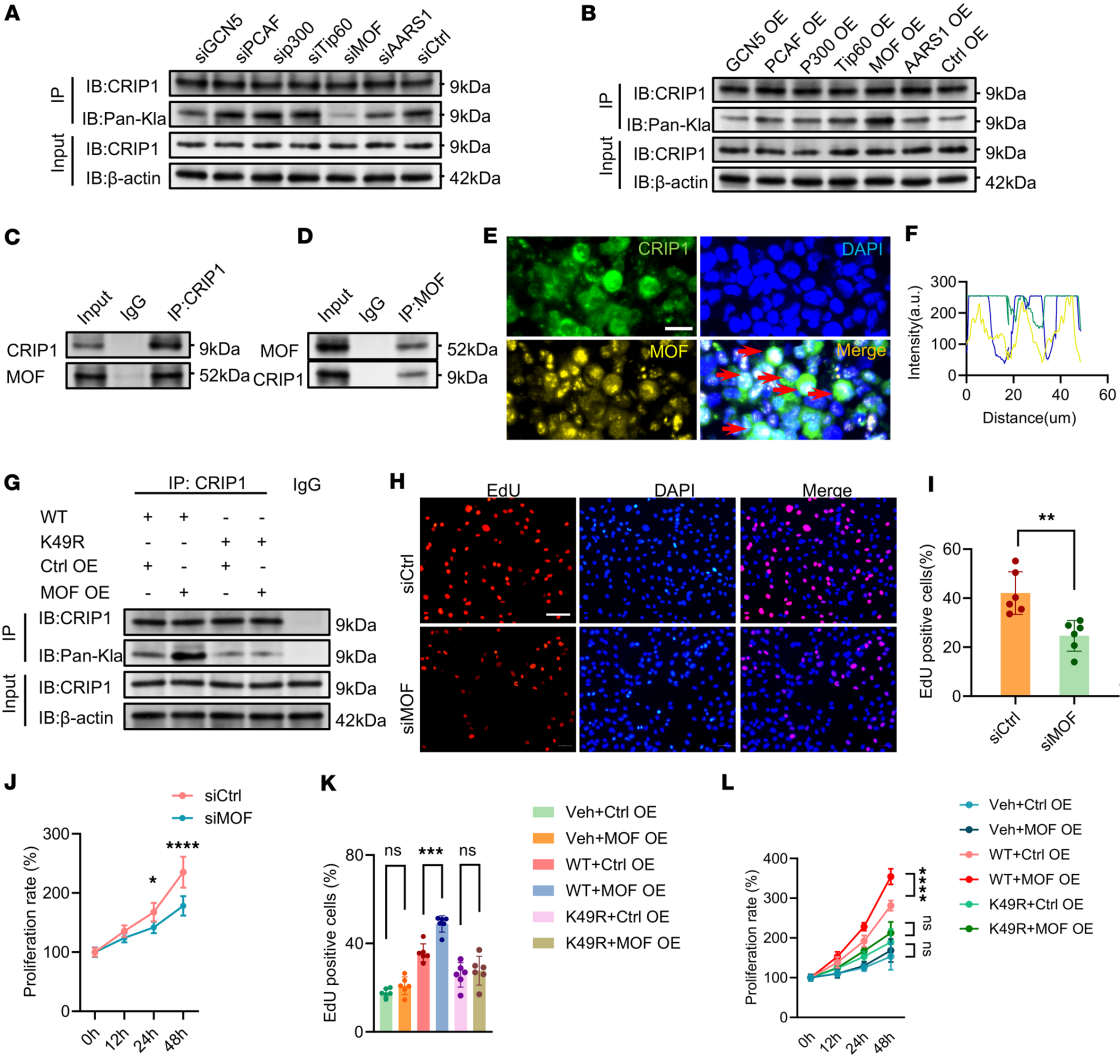
MOF mediates CRIP1 protein lactylation and synovial proliferation in RA. (**A**) Co-IP of CRIP1 followed by immunoblotting for Pan-Kla to detect CRIP1 protein lactylation in RA-FLSs after knockdown of lysine acetyltransferases (GCN5, PCAF, p300, Tip60, MOF, AARS1) (*n* = 3). (**B**) Co-IP of CRIP1 followed by immunoblotting for Pan-Kla to detect CRIP1 protein lactylation in RA-FLSs upon overexpression of lysine acetyltransferases (GCN5, PCAF, p300, Tip60, MOF, AARS1) (*n* = 3). (**C** and **D**) Co-IP assays to detect the interaction between CRIP1 and MOF (*n* = 3). (**E** and **F**) Immunofluorescence staining and quantification of CRIP1 (green) and MOF (yellow) in RA-FLSs. Scale bar: 20 μm. (**G**) Co-IP of CRIP1 followed by immunoblotting for Pan-Kla to detect CRIP1 protein lactylation in RA-FLSs expressing WT or K49R mutant. The assay was performed with or without MOF overexpression. (**H** and **I**) EdU incorporation and quantification of cell proliferation in RA-FLSs after MOF knockdown (*n* = 6). Scale bar: 100 μm. (**J**) CCK-8 assay evaluating proliferation in RA-FLSs after MOF knockdown (*n* = 6). (**K** and **L**) EdU incorporation and CCK-8 assay to quantify cell proliferation in RA-FLSs expressing Veh, WT, or CRIP1 K49R. These assays were performed with or without MOF overexpression (*n* = 6). **P < 0.01, ***P < 0.001, ****P < 0.0001. Data are shown as mean ± SEM and *P*-values are calculated using unpaired 2-tailed *t* test (**I**), or 2-way ANOVA with Bonferroni’s post hoc test (**J** and **L**), or 1-way ANOVA followed by Tukey’s post hoc test (**K**).

To identify the lysine lactyltransferase responsible for CRIP1 K49 lactylation in RA-FLSs, we systematically screened 6 candidate enzymes (GCN5, PCAF, p300, Tip60, MOF, and AARS1) in RA-FLSs. Transfection efficiency was validated at both the mRNA and protein levels (Supplemental Figure 7 and Supplemental Figure 8, A–L). Among the candidates, MOF knockdown led to a striking reduction in CRIP1 K49 lactylation, while overexpression of MOF markedly enhanced this modification (Figure 5, A and B, and Supplemental Figure 8, M and N), suggesting that MOF is a key upstream regulator of CRIP1 protein lactylation. To support, Co-IP assays confirmed the physical association between these molecules (Figure 5, C and D). Immunofluorescence imaging visually depicted the colocalization of MOF and CRIP1 in RA-FLSs (Figure 5, E and F). Of note, MOF overexpression upregulated CRIP1 protein lactylation in CRIP1 WT, while this effect was abolished in the K49R mutant (Figure 5G). Further analysis using EdU and CCK-8 assays revealed that silencing MOF significantly reduced RA-FLS proliferation (Figure 5, H–J), while MOF overexpression promoted proliferation — an effect absent in the vehicle and K49R mutant (Figure 5, K and L). In consequence, MOF is identified as the primary enzyme responsible for CRIP1 protein lactylation in RA-FLSs.

### CRIP1 protein lactylation hijacks p21 from CDK2 to promote synovial proliferation.

**Figure 6:**
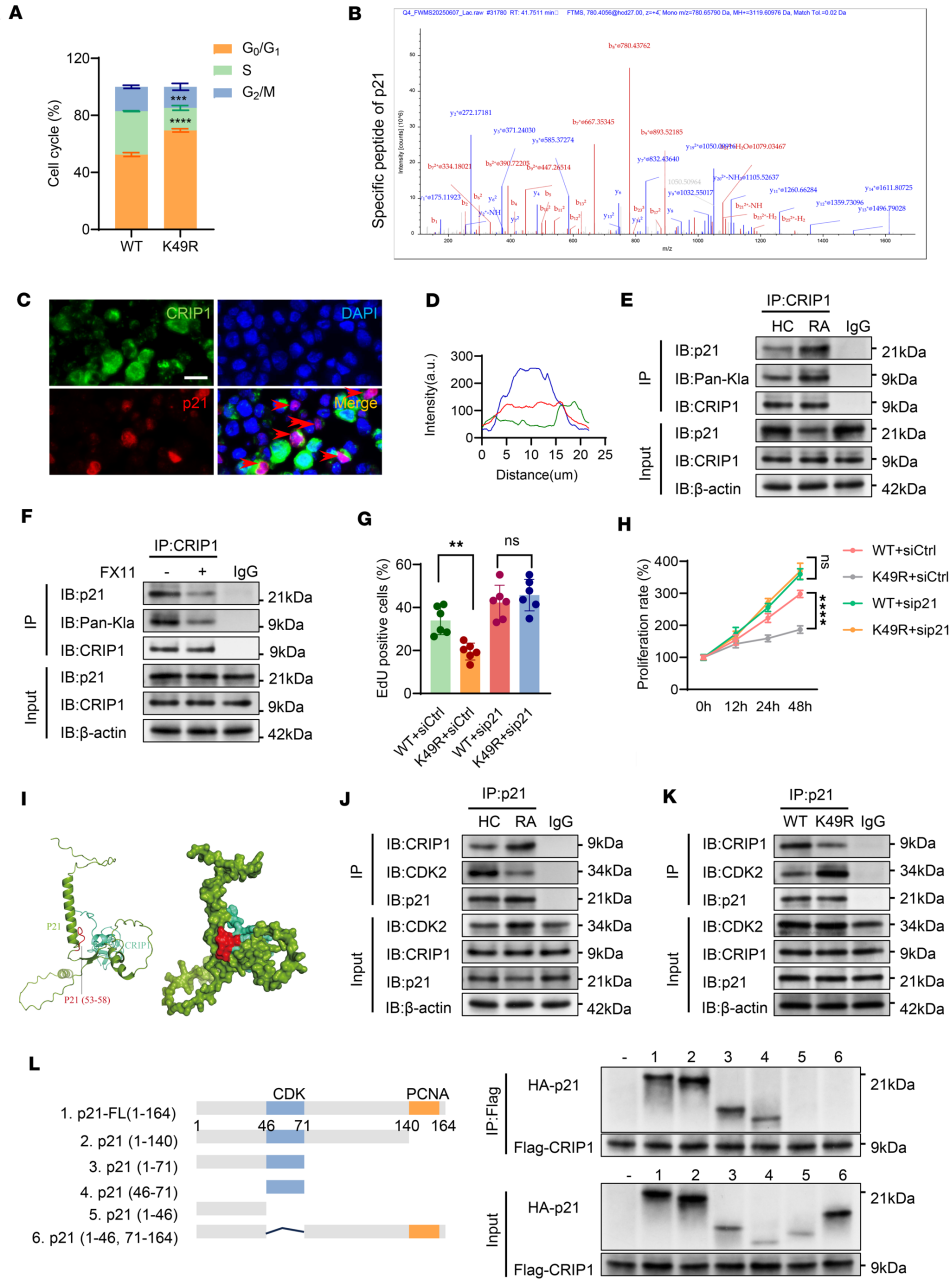
CRIP1 protein lactylation affects p21-CDK2 axis to promote synovial proliferation. (**A**) Flow cytometry analysis of the cell cycle in RA-FLSs (*n* = 3). (**B**) MS/MS spectrum confirming p21 as a binding protein of CRIP1. (**C** and **D**) Immunofluorescence staining and quantification of CRIP1 (green) and p21 (red) in RA-FLSs (*n* = 3). Scale bar: 20 μm. (**E**) Co-IP of CRIP1 followed by immunoblotting for p21 and Pan-Kla to detect CRIP1 protein lactylation and the interaction between CRIP1 and p21 in FLSs (*n* = 3). (**F**) Co-IP of CRIP1 followed by immunoblotting for Pan-Kla and p21 to assess its lactylation and interaction with p21 (20 μM) (*n* = 3). (**G**) EdU incorporation assays to quantify cell proliferation (*n* = 6). (**H**) CCK-8 assay to evaluate proliferation in RA-FLSs (*n* = 6). (**I**) Structural modeling illustrating the predicted interaction interface between CRIP1 and p21. (**J**) Co-IP of p21 followed by immunoblotting for CDK2 and CRIP1 to detect interactions between p21 and CRIP1, as well as between p21 and CDK2 in FLSs. (**K**) p21 was immunoprecipitated and analyzed by immunoblotting for CDK2 and CRIP1 to detect interaction between p21 and CRIP1, as well as between p21 and CDK2 in RA-FLSs. (**L**) Domain mapping of CRIP1-binding region with p21 (left panel). HEK293T cells were cotransfected with Flag-tagged CRIP1 and HA-tagged p21 constructs containing various combinations of amino acid regions. Co-IP was performed using anti-Flag antibody, followed by immunoblotting for HA-p21 (right panel). **P < 0.01, ***P < 0.001, ****P < 0.0001. Data are shown as mean ± SEM and *P* values are calculated using unpaired 2-tailed *t* test (**A**), 1-way ANOVA followed by Tukey’s post hoc test (**G**), 2-way ANOVA with Bonferroni’s post hoc test (**H**). The quantifications for **E** and **F** are presented in Supplemental Figure 9.

To explore how CRIP1 protein lactylation contributes to synovial proliferation, we conducted a series of cell cycle–related assays. Flow cytometry experiments revealed that the CRIP1 K49R mutant led to a G1/S phase arrest in RA-FLSs (Figure 6A). This suggests that CRIP1 protein lactylation directly controls cell cycle in RA-FLSs.

Since CRIP1 harbors a LIM domain typically involved in protein-protein interactions, immunoprecipitation-mass spectrometry was employed to systematically identify its interaction proteins (Supplemental Figure 9A). From the identified molecules, we prioritized those linked to cell cycle. p21 was utilized for further study due to its well-established role as a CDKs inhibitor (Supplemental Figure 9B and Figure 6B). EdU analysis further demonstrated that zinc binding did not mediate the pro-proliferative effect in RA-FLSs (Supplemental Figure 9C). Next, immunofluorescence analysis showed the colocalization of CRIP1 and p21 (Figure 6, C and D). Co-IP analysis validated the physical interaction between CRIP1 and p21. In patients with RA, CRIP1 protein lactylation was elevated, accompanied by an upregulation in its binding to p21 (Figure 6E and Supplemental Figure 9, D and E). Conversely, FX11 diminished CRIP1 protein lactylation, which in turn reduced its interaction with p21 (Figure 6F and Supplemental Figure 9, F and G). Meanwhile, p21 knockdown abolished the inhibitory effect of CRIP1 K49R on RA-FLS proliferation (Figure 6, G and H). These observations indicate that p21 is fundamental to the effect of CRIP1 protein lactylation on RA-FLS proliferation.

To complement corroborate the above findings, molecular docking analysis of was utilized to model the 3D interaction between CRIP1 and p21. The lactylation site K49 was mapped to the C terminal region of the LIM domain in CRIP1 (Supplemental Figure 9H). Notably, residues 53–58 within the CDK domain of p21 was shown to form the direct interface for CRIP1 binding in the predicted complex (Figure 6I). Thus, we supposed that CRIP1 binds to the CDK domain of p21, thereby influencing cell proliferation, and that CRIP1 protein lactylation may strengthen this interaction. When immunoprecipitated with p21, p21/CRIP1 binding enhanced in RA samples; as a result, the p21/CDK2 axis was impaired (Figure 6J). In contrast, the lactylation-deficient CRIP1 K49R restored the p21/CDK2 axis in RA-FLSs (Figure 6K). Truncated variants of p21 were constructed and transfected into HEK293T cells. Upon immunoprecipitation with CRIP1, only those containing the CDK domain were detected by immunoblotting (Figure 6L). Notably, despite undergoing lactylation, the K49-peptide showed no detectable binding to p21 (Supplemental Figure 9, I and J), a finding consistent with its functional role as a competitive inhibitor of full-length CRIP1 lactylation. Collectively, CRIP1 protein lactylation disrupts the p21/CDK2 axis, ultimately facilitating aberrant proliferation in RA-FLSs.

### Targeting CRIP1 protein lactylation inhibits synovial proliferation and alleviates disease progression in RA models.

**Figure 7:**
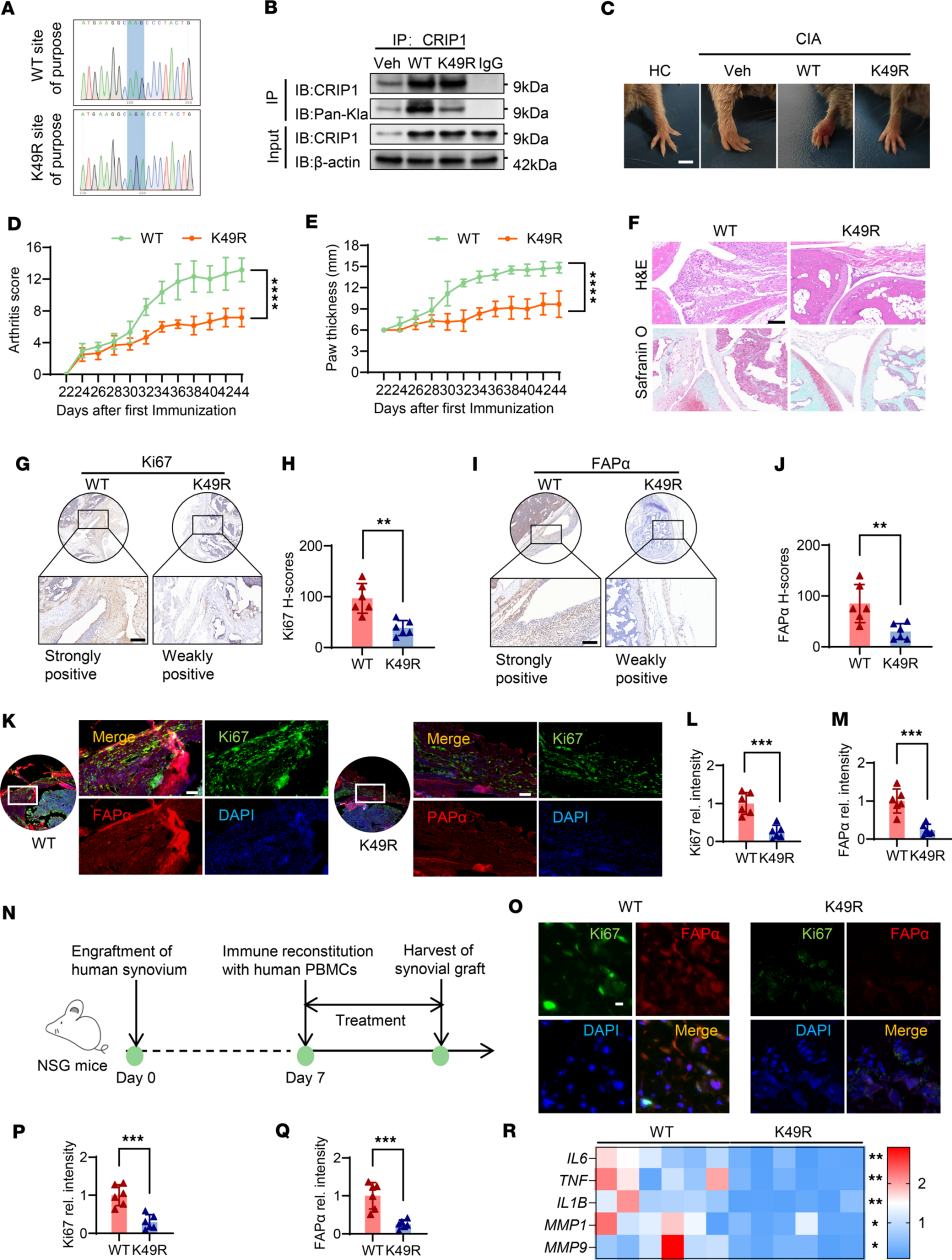
Inhibiting CRIP1 protein lactylation suppresses synovial proliferation and alleviates disease progression in RA disease models. (**A**) Sanger sequencing of the AAG to AGG substitution at the K49 locus in CRIP1. (**B**) CRIP1 was immunoprecipitated and analyzed by immunoblotting for Pan-Kla to detect CRIP1 protein lactylation in synovial tissues. (**C**) Images of forepaws from HC, Veh-treated, WT, or CRIP1 K49R CIA mice. Scale bar: 5 mm. (**D**) Evaluation of clinical arthritis scores in WT or CRIP1 K49R CIA mice (*n* = 6). (**E**) Paw thickness (*n* = 6). (**F**) Histological staining with H&E and Safranin O in synovial tissues (*n* = 6). Scale bar: 100 μm. (**G** and **H**) IHC staining and quantification of Ki67 in synovial tissues from WT or CRIP1 K49R CIA mice (*n* = 6). Scale bars: 100 μm (**I** and **J**) Immunohistochemical staining and quantification of FAPα in synovial tissues from WT or CRIP1 K49R CIA mice (*n* = 6). (**K**–**M**) Immunofluorescence staining and quantification of Ki67 (green), FAPα (red) in synovial tissues from WT or CRIP1 K49R CIA mice. Scale bar: 50 μm. (**N**) Establishment of a human synovial-NSG chimera. NSG mice were implanted with human synovial tissue and reconstituted with PBMCs to generate a humanized RA model. Synovial grafts were subsequently collected to evaluate treatment effects. (**O**–**Q**) Immunofluorescence staining and quantification of Ki67 (green) and FAPα (red) in synovial grafts derived from WT and CRIP1 K49R groups. Scale bar: 10 μm. (**R**) Heatmap depicting mRNA expression levels of inflammation-related genes (*IL6*, *TNF*, *IL1B*) and matrix-degrading enzymes (*MMP1*, *MMP9*) in synovial grafts derived from WT and CRIP1 K49R groups (*n* = 6). *P < 0.05, **P < 0.01, ***P < 0.001, ****P < 0.0001. Data are shown as mean ± SEM and *P* values are calculated using 2-way ANOVA with Bonferroni’s post hoc test (**D** and **E**), or unpaired 2-tailed *t* test (**H**, **J**, **L**, **M**, and **P**–**R**).

The translational relevance of our findings was validated in vivo using a well-established CIA mouse model from DBA/1 mice. We injected AAV particles expressing WT or CRIP1 K49R intraarticularly into the knee joints of CIA mice (Figure 7, A and B). Mice in CRIP1 K49R group showed significantly reduced arthritis symptoms compared with WT CRIP1 group, as evidenced by clinical scores and decreased paw thickness (Figure 7, C–E). Histological assessment brought to reduction in synovial hyperplasia and tissue damage in these mice (Figure 7F). Meanwhile, both IHC and immunofluorescence analysis showed a markedly decrease in Ki67 and FAPα expression, indicating suppressed proliferation and FLSs activation (Figure 7, G–M). Subsequently, we generated humanized RA chimeras implanting clinical synovial tissues into NSG mice for 1 week, followed by injection of human peripheral blood mononuclear cells (PBMCs) from patients with RA (Figure 7N). Consistently, human grafts from mice injected with CRIP1 K49R showed a decrease in the proliferative phenotype compared with CRIP1 WT (Figure 7, O–Q), with reduced inflammation factors (IL-6, TNF, IL-1B) and matrix metalloproteinases (MMP1, MMP9) (Figure 7R).

### A synthetic peptide targeting CRIP1 lactylation reduces disease severity in RA models.

**Figure 8:**
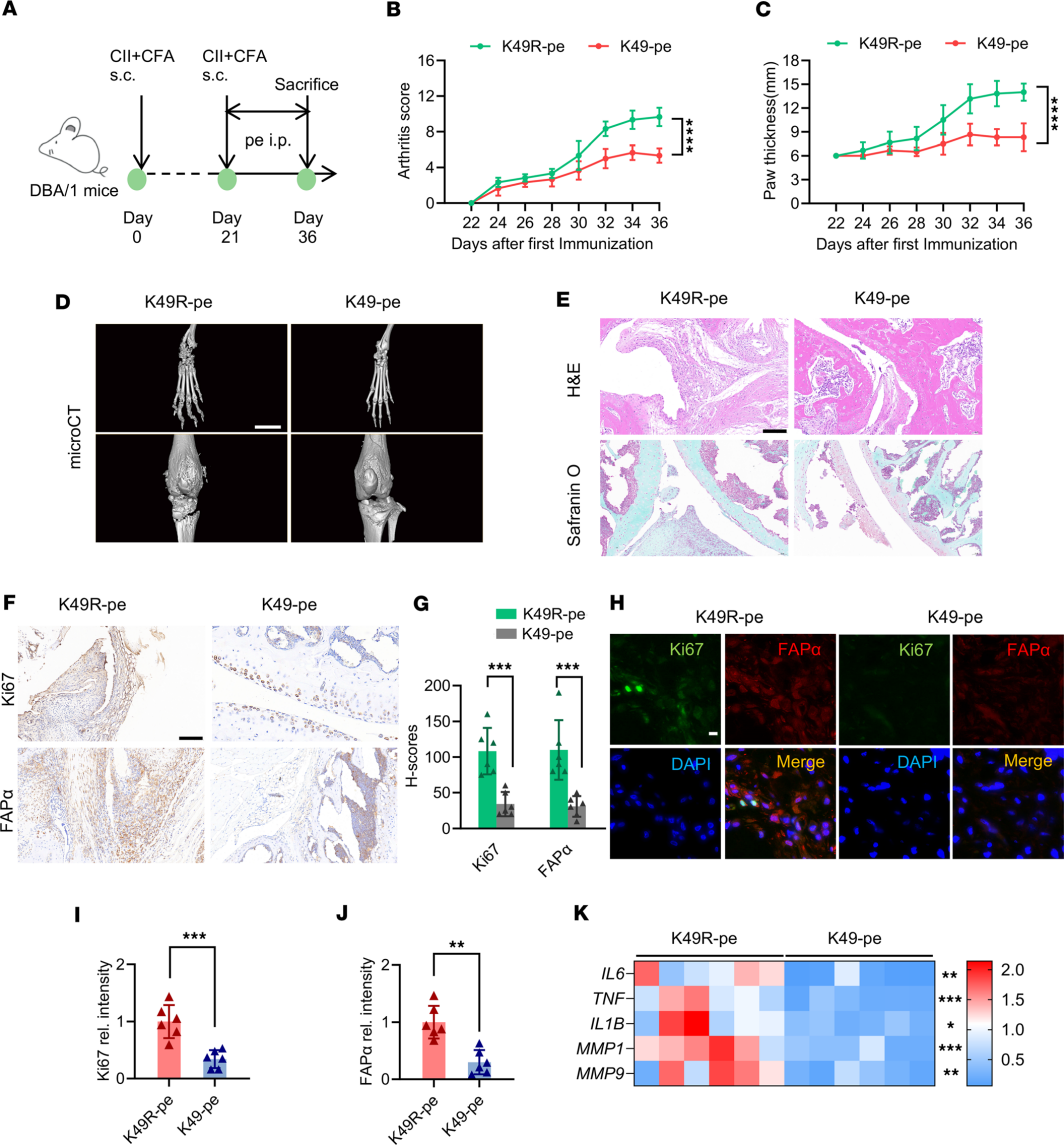
Targeting CRIP1 lactylation with a synthetic peptide suppresses RA progression in mouse models. (**A**) Peptide treatment in CIA mice. (**B**) Evaluation of clinical arthritis scores in K49R peptide– or K49 peptide–treated CIA mice (*n* = 6). (**C**) Paw thickness in K49R peptide– or K49 peptide–treated CIA mice (*n* = 6). (**D**) μCT images of forepaws and knee joints from K49R peptide– or K49 peptide–treated CIA mice. Scale bar: 5 mm. (**E**) Histological staining of H&E and Safranin O in synovial tissues with from K49R peptide– or K49 peptide–treated CIA mice. Scale bar: 100 μm. (**F** and **G**) IHC staining and quantification of Ki67 and FAPα in synovial tissues from K49R peptide– or K49 peptide–treated CIA mice (*n* = 6). Scale bar: 100 μm. (**H**–**J**) Immunofluorescence staining and quantification of Ki67 (green) and FAPα (red) in synovial grafts derived from K49R peptide and K49 peptide groups (*n* = 6). Scale bar: 10 μm. (**K**) Heatmap depicting mRNA expression levels of inflammation-related genes (*IL6*, *TNF*, *IL1B*) and matrix-degrading enzymes (*MMP1*, *MMP9*) in synovial grafts derived from K49R peptide and K49 peptide groups (*n* = 6). *P < 0.05, **P < 0.01, ***P < 0.001, ****P < 0.0001. Data are shown as mean ± SEM and P values are calculated using 2-way ANOVA with Bonferroni’s post hoc test (**B** and **C**), or unpaired 2-tailed t test (**G** and **I**–**K**).

The K49-peptide exhibited therapeutic effects in the aforementioned cell models. Next, we investigated the in vivo effects of the CRIP1 K49 peptide (Figure 8A). In safety assessments, the peptide did not significantly affect body weight (Supplemental Figure 10A), nor did it cause noticeable organ damage (Supplemental Figure 10, B–G). In CIA mice, i.p. injection of the K49 peptide led to a time-dependent reduction in arthritis severity and histopathological damage (Figure 8, B and C). At the molecular level, synovial tissues from these mice exhibited reduced expression of Ki67 and FAPα (Figure 8, D–G). Furthermore, experiments in humanized NSG mice revealed attenuation of the proliferative phenotype (Figure 8, H–J), accompanied by lower levels of inflammatory factors (IL-6, TNF, IL-1B) and matrix metalloproteinase production (MMP1, MMP9) (Figure 8K). Altogether, targeting CRIP1 protein lactylation effectively alleviates excessive proliferation in the synovium of RA mouse model.

## Discussion

FLSs play a central role in the pathogenesis of RA progression (11). Under physiological conditions, these stromal cells were located within synovial lining and sublining layers, supporting immune responses and tissue homeostasis (35). In RA, however, FLSs abandon their quiescent phenotype and undergo a transformation “from friend to foe,” collectively leading to synovium hyperplasia and tissue destruction (8). In the current study, we identify lactate-mediated modification as a key driver of synovial hyperplasia, reinforcing the emerging importance of PTMs in RA pathology. Mechanistically, MOF-induced CRIP1 protein lactylation disrupts the p21/CDK2 axis, advancing the cell cycle and positioning this pathway as a promising therapeutic target for RA intervention.

RA is marked by dramatic hyperplasia of the synovial lining fibroblast layer, progressing from its normal thickness of 1–2 cells to over 10, while sublining fibroblasts show even more extensive expansion (36, 37). Due to their substantial expansion, FLSs have long been considered an attractive therapeutic target in patients with refractory RA (38). Most research in this area has focused on the regulation of CDKs, which govern the transition from G1 to S phase. Initial reports indicate CDK inhibitor p21 reduces IL-6 and MMP1 production in RA-FLSs (39). In a phase 1b trail, seliciclib demonstrates therapeutic potential in TNF inhibitor-refractory patients with RA (40). While prior work on cell cycle control has primarily centered on phenotypic alterations, herein, we offer a more in-depth exploration of the underlying mechanisms in RA-FLSs. To date, there are no approved treatments that directly target FLS proliferation. Thus, our findings could provide a complementary strategy for patients with limited responses to immunotherapies.

Metabolomic profiling has revealed significant perturbations in the RA synovium (41, 42). Indeed, glucose metabolism is globally enhanced, with both the pentose phosphate pathway (PPP) and glycolysis being involved (13). It is well established that activated RA-FLSs correlate with increased glucose uptake, enhanced glycolytic flux, and excessive lactate production (6). Lactate has been identified as the predominant carbon-containing metabolite (86.12%) secreted by RA-FLSs. Elevated glycolytic enzyme activity, lactate secretion, and oxidative stress are positively correlated with diseases severity (43–45). Proliferative RA-FLSs express high levels of lactate transporter MCT4, while the mRNA levels of other transporters, such as Na^+^/H^+^ exchanger 1 (NHE-1) and vacuolar-type H^+^-ATPase (V-ATPase), show no significant changes (18). Hypoxia-inducible factor 1α (HIF-1α) is elevated in patients with RA and directly regulates MCT4 expression (12, 19). Besides, lactate produced by RA-FLSs has been shown to function as a signaling molecule for various immune cells (such as T cells, macrophages, and dendritic cells), modulating their differentiation and activation (17, 45, 46). In this study, we observe a marked increase in glycolytic enzyme LDHA and its product, lactate. Further investigation reveals that lactate accumulation directly induces synovial proliferation in RA. In line with previous studies, our findings underscore the critical importance of glycolytic reprogramming, while also supporting a functional role for lactate metabolism in the progression of RA.

With glucose at the center, metabolites of glycolysis and oxidative metabolism are closely linked to epigenetics and PTMs (47, 48). In RA, widespread abnormal epigenetic modifications occur, particularly in stromal cells (49). Of particular interest, protein citrullination appears to be involved in anticitrullinated protein/peptide antibody–mediated (ACPA-mediated) FLS mobility (50). Epigenetic alterations in RA-FLSs primarily focus on DNA methylation and various histone modifications, which together enhance the expression of genes involved in inflammation, matrix remodeling, and immune responses (51). Lactylation represents an emerging field in PTMs (52). Histone lactylation has been shown to promote FLS invasion and RA progression through an epigenetic/transcriptional axis, characterized by increased NFATc2 expression and the production of antilactylated histone autoantibodies (53). However, the role of lactylation, particularly in nonhistone proteins, remains largely unexplored in RA. In this study, lactylation profiling of human samples provides a comprehensive understanding of this modification in RA. Diverging from metabolic flux–based explanations (54), we uncover a lactate-based PTM mechanism. Specifically, the transfer of the lactyl group from lactate by the acetyltransferase enzyme MOF leads to the lactylation of CRIP1. This modification increases its binding with p21, disrupting the p21/CDK2 axis and consequently promoting synovial proliferation. Our results identify 3 key factors — lactate, MOF, and CRIP1 K49 — as critical regulators of CRIP1 K49 lactylation. Based on this, we propose 3 potential interventions: (a) targeting the aberrant glycolysis pathway using the LDHA inhibitor FX11; (b) pharmacological inhibition with the lactylation writer MOF; and (c) direct therapeutic targeting of CRIP1 K49 residue. A functional CPP was designed to compete with CRIP1 at K49 and block its lactylation. To this end, we synthesized the K49 peptide, which restored cell cycle progression in RA-FLSs. Its translational potential was further validated in RA humanized models that recapitulate the cellular heterogeneity and crosstalk of the RA synovium (55, 56). This peptide may, therefore, offer an option for RA populations with suboptimal responses to current immunotherapies. Consequently, these studies enhance our understanding of how metabolite metabolism influence PTMs in FLSs, providing a foundation for future strategies to selectively target these pathways.

The cysteine-rich intestinal protein (CRIP) family was initially studied for its role in intestinal zinc absorption, a function associated with its LIM domain. Over time, research has expanded to reveal its involvement in a broad range of physiological and pathological processes, including cell proliferation, apoptosis, immunity, and epithelial-mesenchymal transition (EMT) (34, 57, 58). CRIP1 has gained significant attention in cancer due to its context-dependent function as either a suppressor or oncogene. In most solid tumors, it promotes progression through the activation Wnt/β-catenin, Ras/Raf/MEK/ERK, and NF-κB pathways (34, 59, 60). It has also been identified as a potential diagnostic gene biomarker in patients with OA (61). In this study, we identify CRIP1 protein lactylation as a specific contributor to the pathogenesis of RA, rather than a general marker of joint diseases. Previously, the epigenetic regulation of CRIP1 has primarily focused on DNA methylation (62). However, in this work, we uncover the lactylation modification of CRIP1 and characterize its specific modification site. Through an integrated analysis of bioinformatic modeling and biological experiments, we provide evidence that CRIP1 lactylation increases its binding for p21. This underlying mechanism may involve: (a) conformational changes in the protein structure that expose previously hidden binding sites; (b) site-specific modification acting as a signal tag, enabling interactions with regulatory molecules; and (c) the charged lactyl group neutralizing lysine residues, thereby altering the local electrostatic landscape (22).

In the current study, we demonstrate that CRIP1 protein lactylation promotes its binding to p21. However, the precise mechanism remains to be fully explored. Acetylation shares similar enzymatic machinery with lactylation, and the 2 modifications often co-occur on the same proteins or residues. Future research will prioritize examining the acetylation modifications and the PTM crosstalk with lactylation in CRIP1. To investigate the role of delactylation modifications, we have employed AAV injection and CPPs in a humanized RA mouse model. However, the creation of CRIP1 K49 lactylation mutant mice would allow for a more precise means to evaluate the therapeutic effects.

In summary, we have a PTM mechanism in which lactate accumulation-induced CRIP1 protein lactylation acts as a critical switch driving FLS proliferation in RA. These findings not only expand the current understanding of PTM regulation in RA pathogenesis but also position CRIP1 protein lactylation as a promising therapeutic target.

## Methods

### Sex as a biological variable.

In the study with human samples, sex was considered as a biological variable. The RA cohort included 30 female and 10 male participants, consistent with the female predominance of RA. In the mouse experiments, sex was not evaluated as a biological variable, and only male mice were used to minimize the potential effects of estrogen on arthritis-related phenotypes.

### Patients and healthy donors.

Patients with RA were diagnosed according to the 2010 classification criteria established by the American College of Rheumatology and the European Alliance of Associations for Rheumatology (ACR/EULAR). Healthy controls included patients who had undergone trauma surgery and had no clinical history of arthritis. Synovial tissues were collected during joint replacement or synovectomy procedures, while synovial fluid samples were obtained specifically during synovectomies.

### Cell isolation and culture.

Primary FLSs were isolated from synovial tissues and digested with type II collagenase (BioFroxx, 2275MG100) at 37°C for 3 hours. The resulting suspension was filtered through a 70 μm cell strainer and cultured in F12/DMEM supplemented with 10% FBS. The in vitro experiments included 3 major treatment strategies: (a) Metabolic lactylation modulation: Sodium lactate (Nala, Sigma-Aldrich, L7022) was added at final concentrations of 10 mM or 20 mM to induce lactylation. In contrast, a selective LDHA inhibitor (FX11; MCE, HY-16214) was applied at 10 μM or 20 μM to inhibit lactylation. Treatment duration was 48 hours. (b) CRIP1 protein lactylation-targeted gene modulation: FLSs were transduced with lentiviral vectors encoding either CRIP1 WT or a lactylation-deficient mutant CRIP1 K49R (OBIO Technology Co. Ltd.). Lentivirus was applied at a multiplicity of infection (MOI) of 20. (c) Peptide-based intervention: FLSs were exposed for 48 hours with K49 or K49R-peptide at 20 μM. HEK293T cells (ATCC, CRL-3216) were cultured in DMEM supplemented with 10% FBS at 37°C in a humidified incubator with 5% CO_2_.

### Lactate quantification in synovial fluid.

Lactate concentration was determined using a lactate assay kit (Roche Diagnostics, 06343759001), following the manufacturer’s protocol. Absorbance was measured at 552 nm using a Cedex Bio Analyzer (Roche).

### Lysine lactylation–based (Kla–based) PTM enrichment.

Synovial tissues from patients with RA and healthy individuals were pulverized in liquid nitrogen and lysed. After centrifugation (12,000 × *g*, 20 min) and protein quantification, samples were reduced with DTT, alkylated with IAA, and digested with trypsin overnight. Digestion was stopped by acidification. Lactylated peptides were enriched using Pan-Kla antibody–conjugated beads at 4°C overnight, thoroughly washed, eluted, vacuum dried, and desalted with C18 ZipTips (Millipore). Peptides were reconstituted in 0.1% formic acid before LC-MS/MS analysis.

### HPLC-MS/MS analysis.

High-performance liquid chromatography–tandem mass spectrometry (HPLC-MS/MS) was performed with support from Shanghai Applied Protein Technology Co. Ltd. iRT standard peptides were added to each sample before analysis. Data were acquired on an Orbitrap Astral mass spectrometer coupled to a Vanquish Neo UHPLC system (Thermo Scientific) in DIA mode. MS1 scans were collected across m/z 380–980 at a resolution of 240,000 (200 m/z), with a normalized AGC target of 500% and maximum injection time of 5 ms. DIA-MS2 acquisition used 299 variable windows (2 m/z), with HCD collision energy of 25 eV, normalized AGC target of 500%, and maximum injection time of 3 ms. Data were processed with DIA-NN 1.8.1, and lysine lactylation peptide ratios were normalized to protein expression levels.

### Proliferation assays.

Cells were treated with EdU (Beyotime, C0075S), and the percentage of EdU^+^ was calculated. Cell viability was measured using the CCK-8 assay (Cytoch, CP0001), with absorbance measured at 450 nm. All procedures were performed according to the manufacturer’s instructions.

### Gene silencing and overexpression.

Cells were transfected with siRNAs (OBIO Technology) or plasmids using Lipofectamine 3000 Transfection Reagent (Invitrogen, L3000008). Knockdown and overexpression efficiencies were assessed at the mRNA and protein levels by qPCR and Western blotting. The siRNA sequences are provided in Supplemental Table 2.

### Western blot.

Total protein was extracted using RIPA buffer. Equal amounts of protein were denatured in 1× SDS loading buffer, separated by SDS-PAGE, and transferred to PVDF membranes. The membranes were blocked and incubated with primary antibodies, followed by HRP-conjugated secondary antibodies. The primary antibodies used included Pan-Kla (PTM Bio, 1401RM), LDHA (Proteintech, 19987-1-AP), COL1A1 (Proteintech, 67288-1-Ig), CILP (Abcam, ab192881), CNN3 (Proteintech, 11509-1-AP), AHNAK (Proteintech, 16637-1-AP), CRIP1 (Proteintech, 15349-1-AP), GCN5 (Proteintech, 66575-1-Ig), PCAF (Proteintech, 28770-1-AP), p300 (Proteintech, 20695-1-AP), Tip60 (Proteintech, 10827-1-AP), AARS1 (Proteintech, 17394-1-AP), MOF (Proteintech, 13842-1-AP), and β-actin (Proteintech, 20536-1-AP).

### Co-IP.

Cells were lysed in IP buffer with protease inhibitors, and lysates were cleared by centrifugation (12,000 × *g*, 10 min). The supernatants were incubated with primary antibodies, followed by addition of prewashed protein A/G magnetic beads to capture immune complexes. After extensive washing with cold lysis buffer, proteins were eluted in SDS loading buffer, and the eluted samples were analyzed by SDS-PAGE and immunoblotting or mass spectrometry. The following primary antibodies were used: CRIP1 (Proteintech, 15349-1-AP), Pan-Kla (PTM Bio, PTM-1401RM), MOF (Proteintech, 13842-1-AP), p21 (Abcam, ab109520), CDK2 (Proteintech, 10122-1-AP), Biotin (Abcam, ab1227), HA-Tag (Cell Signaling, 3724), Flag-Tag (Cell Signaling, 2368), and IgG control (Proteintech, 30000-0-AP).

### In vitro lactylation assay.

Bio-tagged peptides were incubated with MOF proteins (MCE, HY-P701625) in reaction buffer containing 50 mM HEPES (pH 7.8), 30 mM KCl, 5 mM MgCl_2_, 5 mM sodium butyrate, 0.25 mM EDTA, 2.5 mM DTT, and 20 μM lactyCoA for 30 minutes at 30°C. The reactions were separated by SDS-PAGE and analyzed by dot blot.

### Tyramide signal amplification (TSA) multiplex immunofluorescence.

For signal amplification, a TSA kit (PerkinElmer) was used according to the manufacturer’s protocol. The following primary antibodies were used: Pan-Kla (PTM Bio, 1401RM), CRIP1 (Proteintech, 15349-1-AP), PDPN (SAB, 49474), Ki67 (Proteintech, 27309-1-AP), MOF (Proteintech, 13842-1-AP), FAPα (Proteintech, 11779-1-AP), p21 (Abcam, ab109520), and LDHA (Proteintech, 19987-1-AP).

### TUNEL assay.

Cells were stained using a TUNEL assay kit (Beyotime, C1086) according to the manufacturer’s instructions. Apoptotic cells were identified as TUNEL^+^.

### Transwell assay.

Cells in serum-free F12/DMEM were seeded in the upper chamber, with 10% FBS in the lower chamber. Transwell assays were performed using 24-well inserts with 8 μm pores (Corning). After 48 hours, migrated cells were fixed, stained with crystal violet, and quantified.

### Single-cell transcriptomic analysis.

To investigate the cellular distribution of CRIP1 expression in synovial tissue, publicly available single-cell RNA-seq (scRNA-seq) data were accessed from the Gene Expression Omnibus (GEO) under accession no. GSE145286 (GEO ID: 200145286). Data visualization and analysis were performed using the Single Cell Portal (https://singlecell.broadinstitute.org/).

### qPCR.

Total RNA was extracted using TRIzol reagent (Invitrogen, 15596026) according to the manufacturer’s instructions. First-strand cDNA was synthesized from 1 μg of total RNA in a 20 μL reaction volume using the FastKing RT Kit (Tiangen, KR116). qPCR was performed on a using SuperReal PreMix Plus (Tiangen, FP205). The thermal cycling conditions were as follows: 95°C for 15 minutes, followed by 40 cycles of 95°C for 10 seconds and 60°C for 30 seconds. Gene expression levels were normalized to ACTB, and relative quantification was determined using the 2^−ΔΔCt^ method. Primer sequences are provided in Supplemental Table 3.

### Flow cytometry.

Cells were collected and stained with PI solution (Multi Sciences, CCS012) following the manufacturer’s instructions. The cells were then promptly analyzed by flow cytometry to assess cell cycle distribution.

### Immunohistochemical staining.

Paraffin-embedded synovial tissue sections were deparaffinized, rehydrated, and subjected to antigen retrieval with a citrate-based buffer under high temperature. Sections were incubated with primary antibodies, followed by HRP-conjugated secondary antibodies. Signal was developed with DAB substrate, and sections were counterstained with hematoxylin for observation. Primary antibodies used: Ki67 (Proteintech, 27309-1-AP), FAPα (Proteintech, 11779-1-AP), and LDHA (Proteintech, 19987-1-AP).

### Molecular docking.

The structure of CRIP1 was obtained from the AlphaFold Protein Structure Database (AF-P50238-F1), and the structure of p21 was retrieved from the Protein Data Bank (PDB ID: 6P8H). Protein-protein docking was performed using the HADDOCK 2.4 web server (https://rascar.science.uu.nl/haddock2.4/). The default parameters and settings were applied for the HADDOCK clustering analysis. PyMOL 3.1.5.1 was used to analyze and generate point mutation and images of the models.

### Peptide synthesis.

All peptides used in this study were synthesized by Guoping Pharmaceutic Inc. (Hefei, China) using standard solid-phase peptide synthesis. Peptides were purified to > 95% purity via HPLC and provided as trifluoroacetate salts. All peptides carried N-terminal acetylation and C-terminal amidation modifications to enhance stability.

### Collagen II–induced arthritis (CIA) model.

Male DBA/1 mice (8 weeks old) were obtained from Cavens Laboratory Animal Co. Ltd. and maintained under specific pathogen–free (SPF) conditions. CIA was induced by emulsifying 10 mg of bovine type II collagen (Chondrex, 20022) in 5 mL of 50 mM acetic acid with an equal volume of Freund’s complete adjuvant (Chondrex, 7001). Mice received 100 μL of the CII emulsion via intradermal injection at the tail base on day 0, followed by a booster injection on day 21. At the end of the experiment, mice were sacrificed, and samples of joint tissue, liver, and kidney were harvested for analysis. Scoring criteria was specified as the following: 0 (no symptoms), 1 (swelling at knuckles), 2 (mild swelling at ankle/wrist), 3 (severe paw swelling), and 4 (joint deformity or ankylosis).

Treatments corresponding to experimental groups were initiated on day 21. CIA mice were randomly assigned to treatment groups (*n* = 6 per group) as follows: (a) Lactate inhibition therapy: i.p. injections of either vehicle control (PBS, 50 μL, Qd) or LDHA inhibitor (FX11, 1 mg/kg, Qd). (b) CRIP1 protein lactylation-targeted gene therapy: Intra-articular injections into the knee joints with adeno-associated virus serotype 5 (AAV5) vectors carrying either WT or CRIP1 K49R (5 × 10¹¹ PFU). (c) Peptide-based therapy: Intraperitoneally injections with K49 or K49R-peptide (0.5 mg/kg, Qd).

### Human synovial tissue-NSG mouse chimeras.

NSG mice (6 weeks old) were obtained from Cavens Laboratory Animal Co. Ltd. and maintained under SPF conditions. Small pieces (4–5 mm in diameter) of human synovial tissues from patients with RA were implanted into the s.c. pocket on the dorsal midline of NSG mice. On day 7 after implantation, the human synovial-NSG chimeras were reconstituted with 1 × 10^7^ PBMCs from patients with RA via i.v. injection. Mice were randomized and assigned to the following treatment groups (*n* = 6 per group), starting on day 7. (a) S.c. injection of AAV5 vectors encoding WT or CRIP1 K49R into synovial explants. On day 28, mice were euthanized, and synovial grafts were harvested for analysis. (b) I.p. injections of K49 or K49R-peptides. On day 14, synovial grafts were harvested for analysis.

### μCT imaging.

Mouse joints were scanned using a μCT system (VNC-102, Phecda Healthcare Technology). Scanning was performed at 90 kV and 540 μA with an exposure time of 200 ms. Three-dimensional reconstructions were generated using Recon software, and image visualization and analysis were performed with Cruiser and Avatar software.

### Histomorphometric analysis.

Paraffin-embedded tissue sections were prepared for histological analysis. H&E staining was utilized to evaluate inflammatory cell infiltration and synovial hyperplasia. Safranin O/Fast Green staining was employed to assess the integrity of the cartilage matrix.

### Statistics.

Statistical analysis was performed using GraphPad Prism 9 (GraphPad Software). Data are presented as means ± SEM. For comparisons between 2 groups, an unpaired Student’s *t* test was used. For multiple-group comparisons, 1-way ANOVA with Tukey’s post hoc test was applied. For experiments with 2 independent variables, 2-way ANOVA with Bonferroni’s post hoc test was used. Correlation was assessed using Pearson’s correlation. *P* < 0.05 was considered statistically significant. All experiments were performed with at least 3 independent biological replicates.

### Study approval.

All human studies were conducted with the approval of the Human Ethics Committee of Wuxi People’s Hospital affiliated with Nanjing Medical University (approval no. KY24138). Appropriate informed consent form was obtained from all participants. Detailed baseline demographic and clinical characteristics of all enrolled participants are summarized in Supplemental Table 1. All animal experiments were approved by the IACUC of Wuxi People’s Hospital affiliated with Nanjing Medical University and were conducted in accordance with institutional and national guidelines for the care and use of laboratory animals (approval no. DL2024024).

### Data availability.

All data relevant to the study are included in the article or uploaded as supplemental information. Data are available in Supporting Data Values file and could be obtained from the corresponding authors upon reasonable request.

## Author contribution

Z Wen, TL, and FY designed and supervised the study. MM, YZ, and QL performed experiments and analyzed data. SG, XW, Z Wang, and HZ collected samples and participated in data analysis. MM and Z Wen wrote the manuscript with input from all authors.

## Conflict of interest

The authors have declared that no conflict of interest.

## Funding support

- National Natural Science Foundation of China (82271841, to ZW)
- Natural Science Foundation of Heilongjiang Province (LH2020H054, to XW)
- Wuxi Municipal Science and Technology Bureau (Y20232009, to FY; K20253017, to TL)
- Wuxi Health Commission General Project (M202320, to TL)
- Medical Center Foundation of Nanjing Medical University (WMCG202519, to YZ; WMCG202413, to HZ)
- Jiangsu Provincial Health Innovation Team
- Priority Academic Program Development of Jiangsu Higher Education Institutions (PAPD)

## Supplementary Material

Supplemental data

Unedited blot and gel images

Supporting data values

## Version 1 06/08/2026

Electronic publication
