# Supplementary material for: Lactate programs CRIP1 protein lactylation to drive synovial proliferation in rheumatoid arthritis
Source: JCI Insight. 2026 Jun 8;11(11):e200928. doi: 10.1172/jci.insight.200928 (PMC13313492; doi:10.1172/jci.insight.200928)
Supplement: Supplemental data [file jciinsight-11-200928-s034.pdf]

Supplementary Figure 1

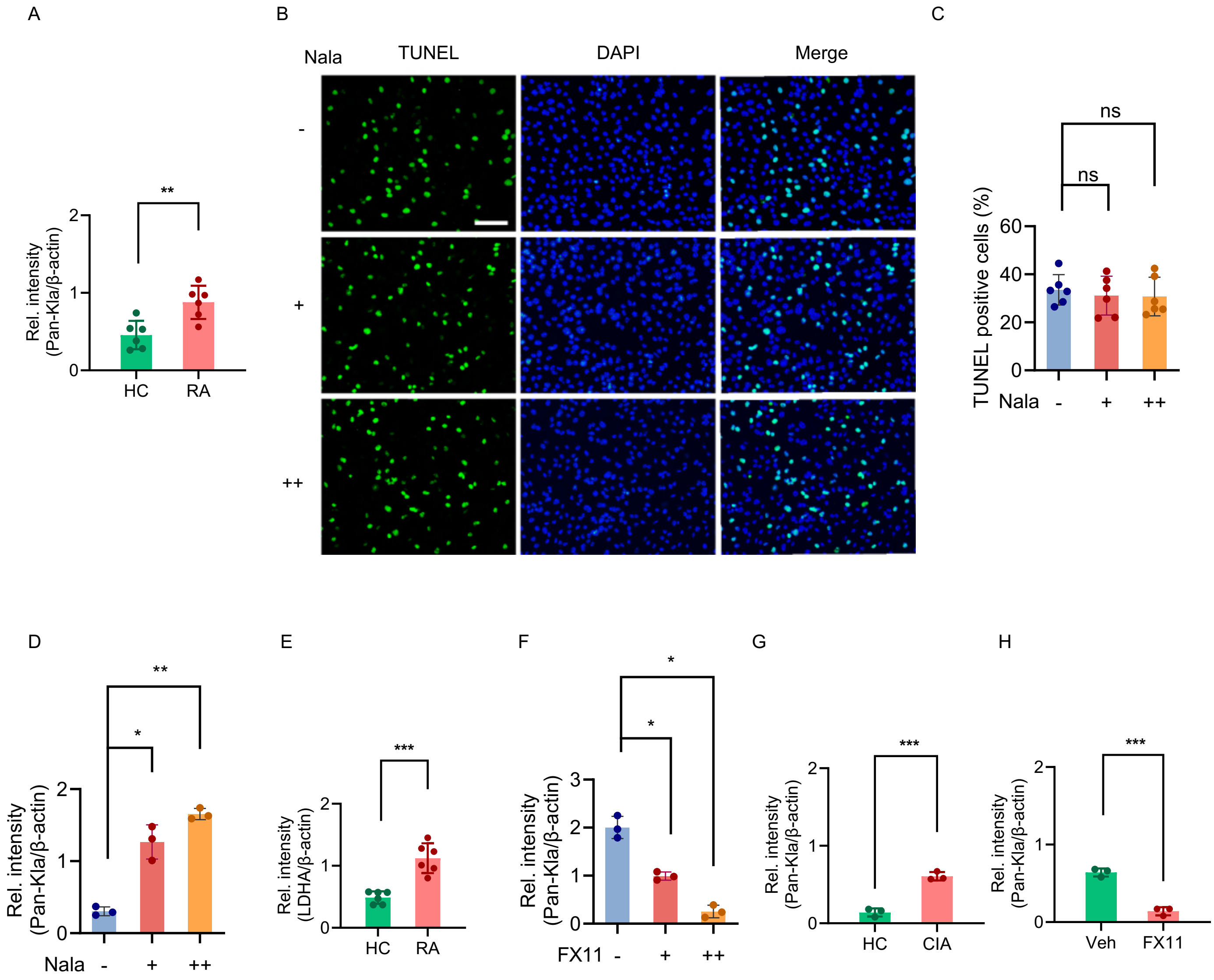

Figure S1. Lactate promotes the synovial proliferation

(A) Quantification analysis in Figure 1H. (**B–C)** TUNEL assay to assess apoptotic cells treated with Nala (10 mM, 20 mM) (n = 6). **(D)** Quantification analysis in Figure 1L. **(E)** Quantification analysis in Figure 2C. **(F)** Quantification analysis in Figure 2J. **(G)** Quantification analysis in Figure 2L. **(H)** Quantification analysis in Figure 2M. Scale bar: 100 μm. ns = no significance, *$p < 0.05$, **$p < 0.01$, ***$p < 0.001$. Data are presented as mean ± SEM, and *p*-values are calculated using unpaired two-tailed t-test (A, E, G, H), or one-way ANOVA followed by Tukey's post-hoc test (C, D, F).

Supplementary Figure 2

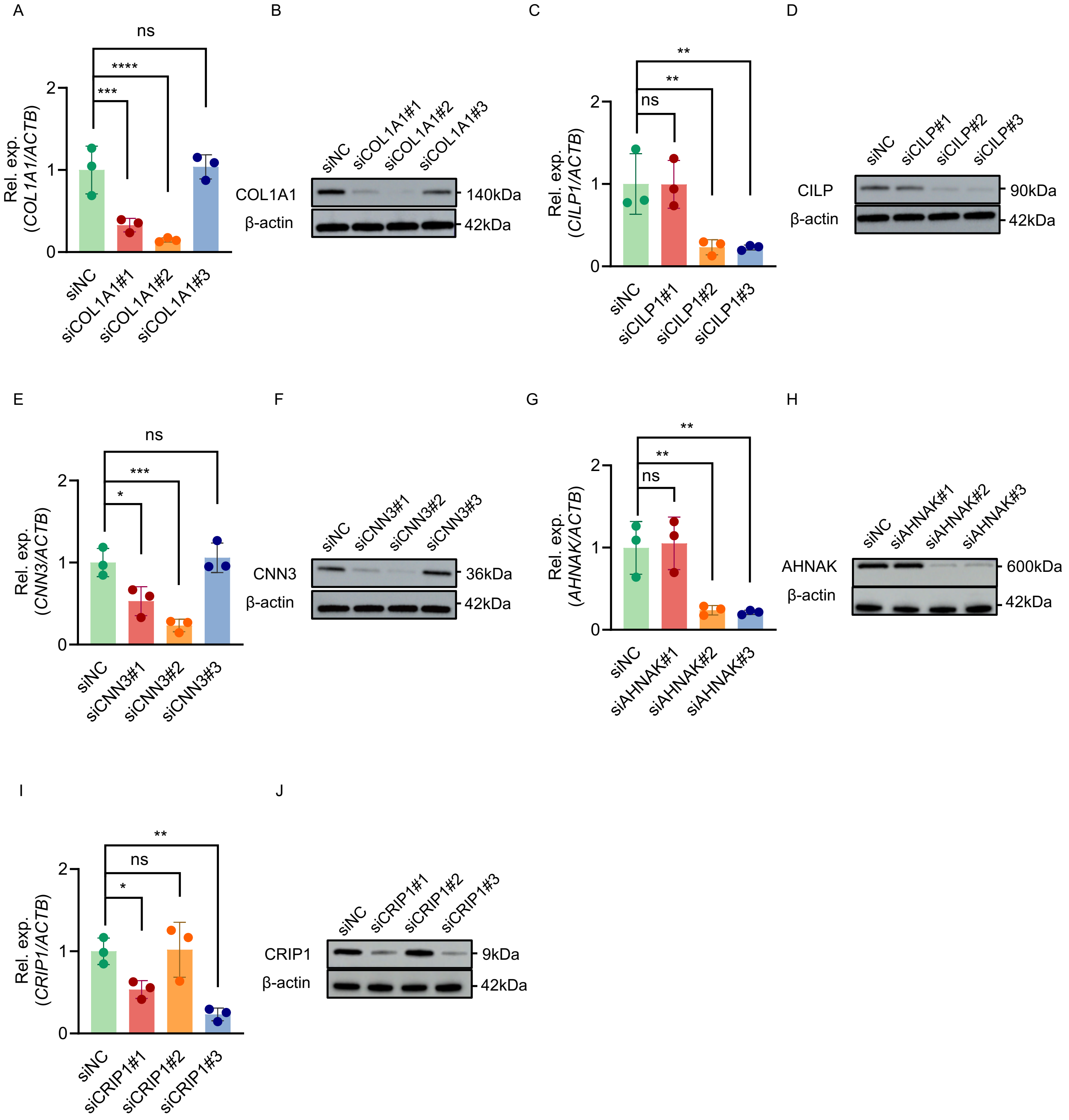

Figure S2. Efficiency validation of lactylation protein knockdown

qRT-PCR to quantify mRNA levels and western blotting to evaluate protein expression in RA-FLSs transfected with siCtrl or siRNA targeting *COL1A1, CILP, CNN3, AHNAK, CRIP1*. ns = no significance, *$p < 0.05$, **$p < 0.01$, ***$p < 0.001$, ****$p < 0.0001$. Data are presented as mean ± SEM, and *p*-values are calculated using one-way ANOVA followed by Tukey's post-hoc test (A, C, E, G, I).

Supplementary Figure 3

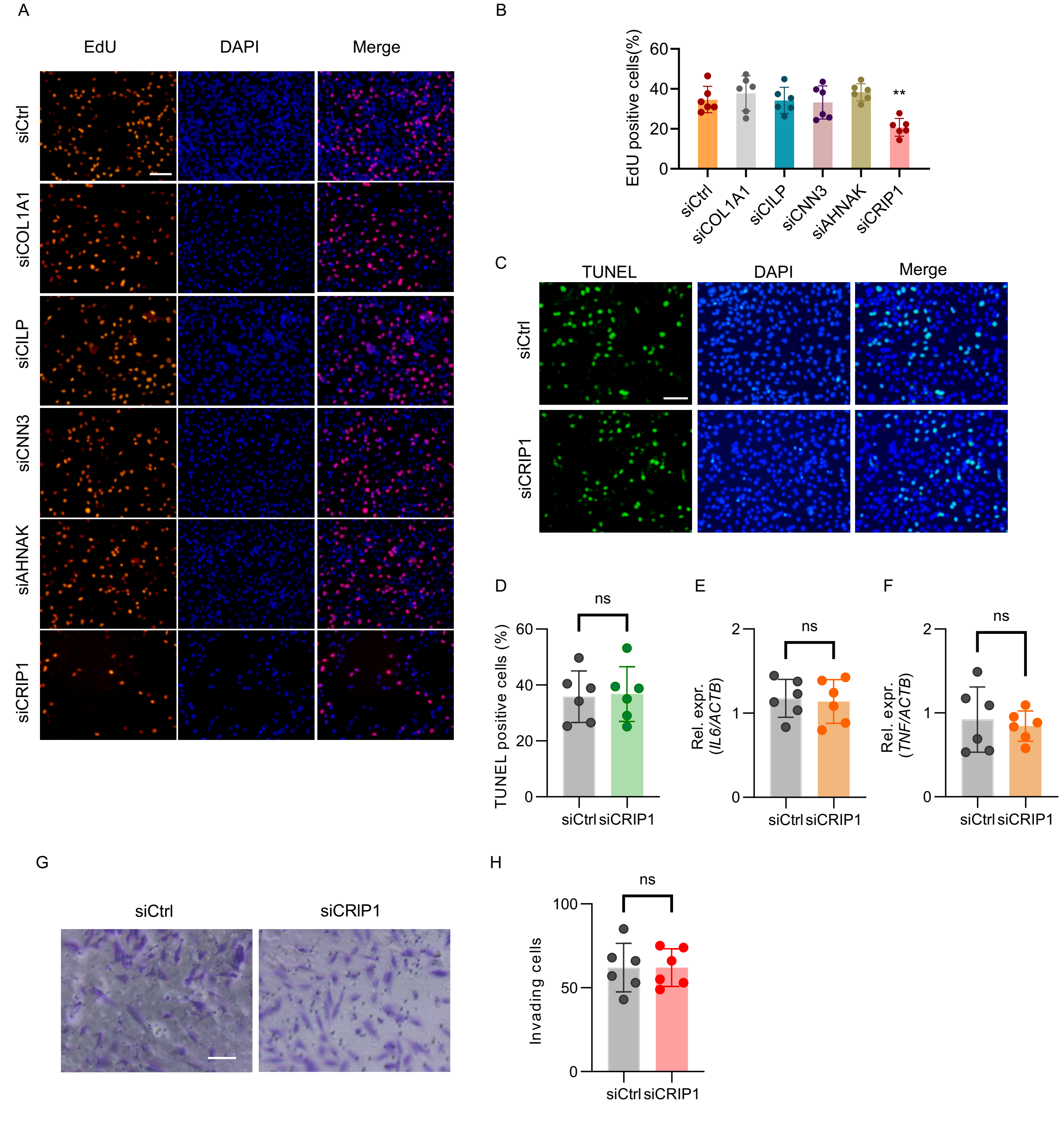

Figure S3. CRIP1 lactylation promotes the proliferation of RA-FLSs

(A–B) EdU incorporation assays and quantification of cell proliferation in RA-FLSs after knockdown of the candidate genes (*COL1A1, CILP, CNN3, AHNAK, CRIP1*). Scale bar: 100 μm. **(C–D)** TUNEL assay to assess apoptotic cells in RA-FLSs after CRIP1 knockdown. Scale bar: 100 μm. **(E–F)** qRT-PCR to quantify mRNA levels of *IL6* and *TNF*. **(G–H)** Transwell assays to measure cell invasion. ns = no significance, $^{**}p < 0.01$. Data are presented as mean ± SEM, and *p*-values are calculated using one-way ANOVA followed by Tukey's post-hoc test (B), or unpaired two-tailed t-test (D, E, F, H).

Supplementary Figure 4

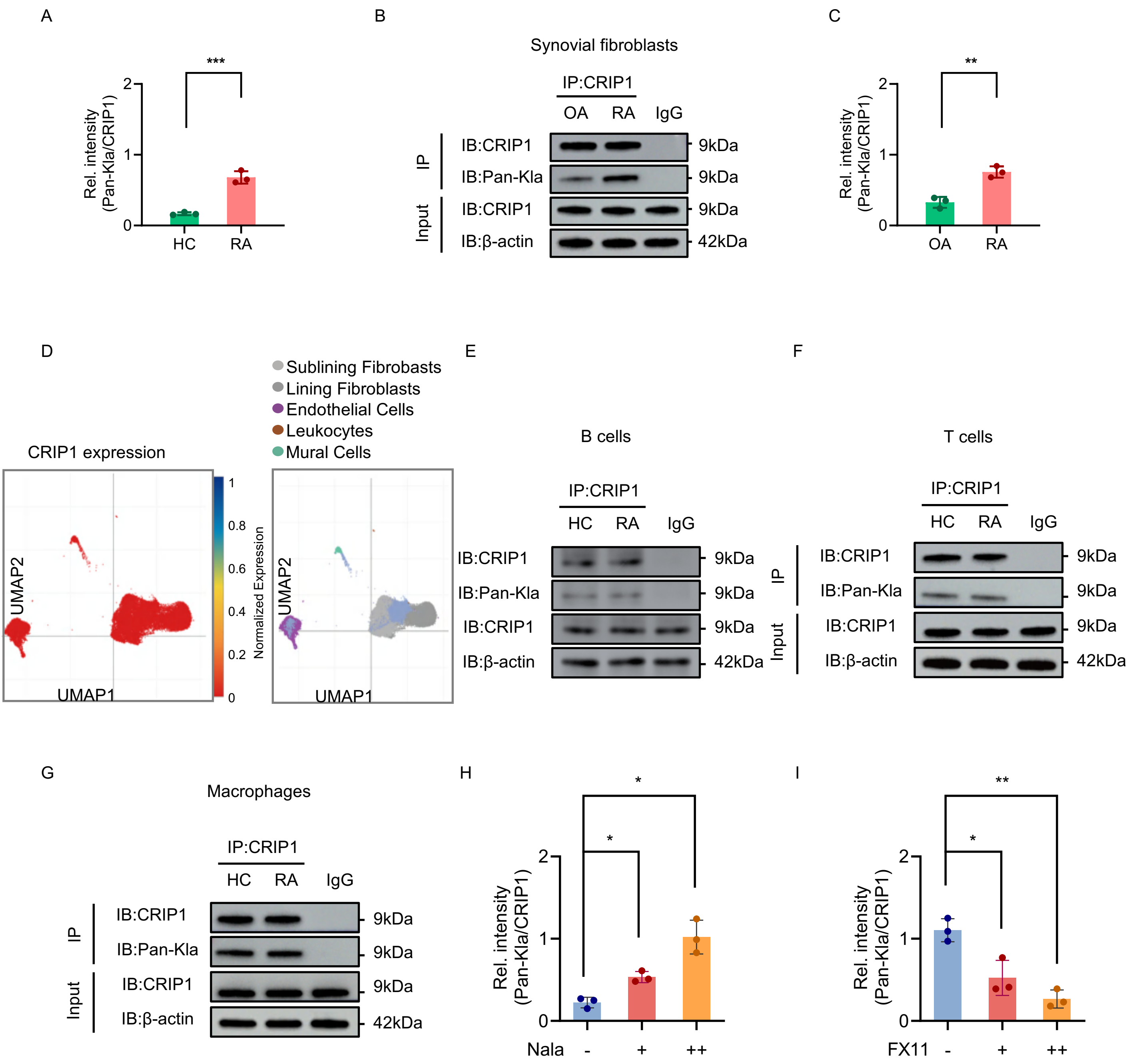

Figure S4. CRIP1 lactylation is elevated in RA-FLSs.

(A) Quantification of Figure 3G. **(B–C)** Co-IP of CRIP1 followed by immunoblotting for Pan-Kla to detect CRIP1 protein lactylation in FLSs from RA and OA patients (n = 3). **(D)** UMAP visualization of scRNA-seq data showing CRIP1 expression across distinct cell populations in synovial tissue. **(E)** Co-IP of CRIP1 followed by immunoblotting for Pan-Kla to detect CRIP1 protein lactylation in B cells from RA patients and healthy controls. **(F)** Co-IP of CRIP1 followed by immunoblotting for Pan-Kla to detect CRIP1 protein lactylation in T cells. **(G)** Co-IP of CRIP1 followed by immunoblotting for Pan-Kla to detect CRIP1 protein lactylation in macrophage. **(H)** Quantification analysis in Figure 3I. **(I)** Quantification analysis in Figure 3J. $^{*}p < 0.05$, $^{**}p < 0.01$, $^{***}p < 0.001$. Data are presented as mean ± SEM, and *p*-values are calculated using unpaired two-tailed t-test (A, C) or one-way ANOVA followed by Tukey's post-hoc test (H, I).

Supplementary Figure 5

A

CRIP1 lactylated modification

Peptide	Site
CNKEVYFAER	(K9)
VTSLGKDWHRPCLK	(K22)
PCLKCEK	(K30)
TLTSGGHAEHEGKPYCNHPCYAAMFGPK	(K49)
GGAESHTFK	(K77)

B

Lactylated modification (K9)

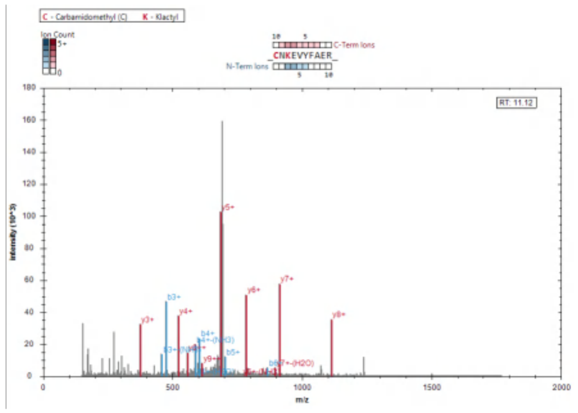

D

Lactylated modification (K30)

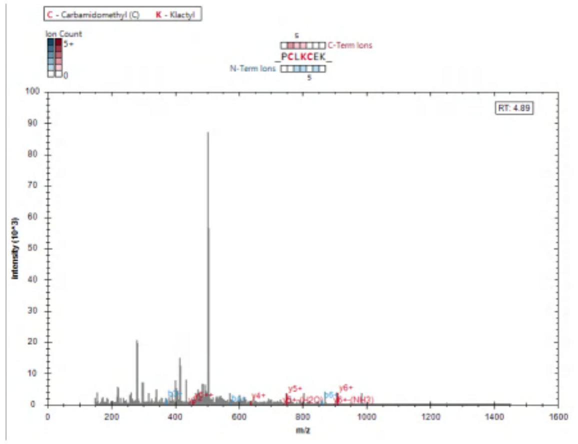

C

Lactylated modification (K22)

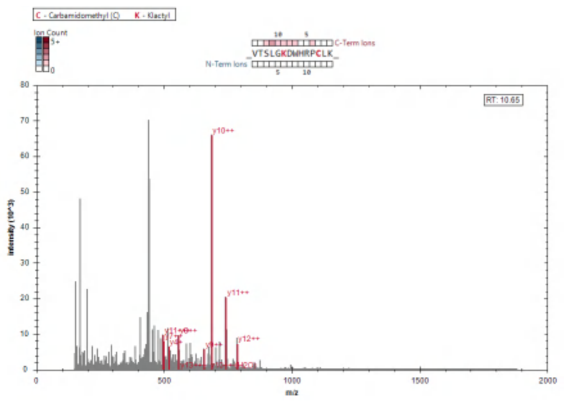

E

Lactylated modification (K77)

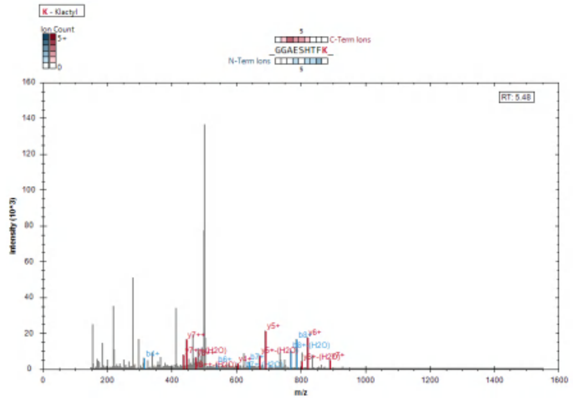

F

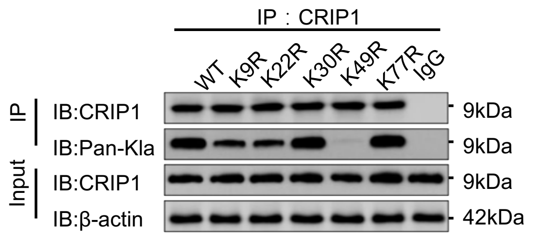

G

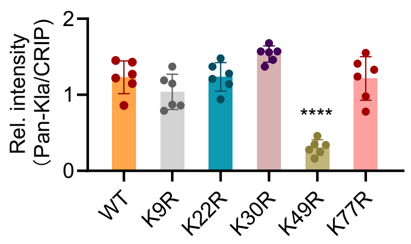

H

Homo sapiens
KTLTSGGHAEHEGKPYCNHPCYAAMFGPKGF
Mus musculus
KTLTSGGHAEHEGKPYCNHPCYSAMFGPKGF
Rattus norvegicus
KTLTSGGHAEHEGKPYCNHPCYSAMFGPKGF
Pan paniscus
KTLTSGGHAEHEGKPYCNHPCYAAMFGPKGF
Aphelocoma coerulescens
KTLTSGGHAEHDGKPYCNHPCYAALFGPKGF

Figure S5. Identification of CRIP1 lactylation sites

(A) Identification of lysine lactylation sites on CRIP1 by LC-MS/MS. **(B–E)** MS/MS spectrum of lysine lactylation in CRIP1. **(F–G)** Co-IP of CRIP1 followed by immunoblotting for Pan-Kla in RA-FLSs expressing WT or its site-specific mutants CRIP1 (K9R, K22R, K49R, and K77R) (n = 6). **(H)** Protein sequence alignment analysis of the CRIP1 K49 site across multiple species. ****$p < 0.0001$. Data are presented as mean ± SEM, and p-values are calculated using one-way ANOVA followed by Tukey's post-hoc test (G).

Supplementary Figure 6

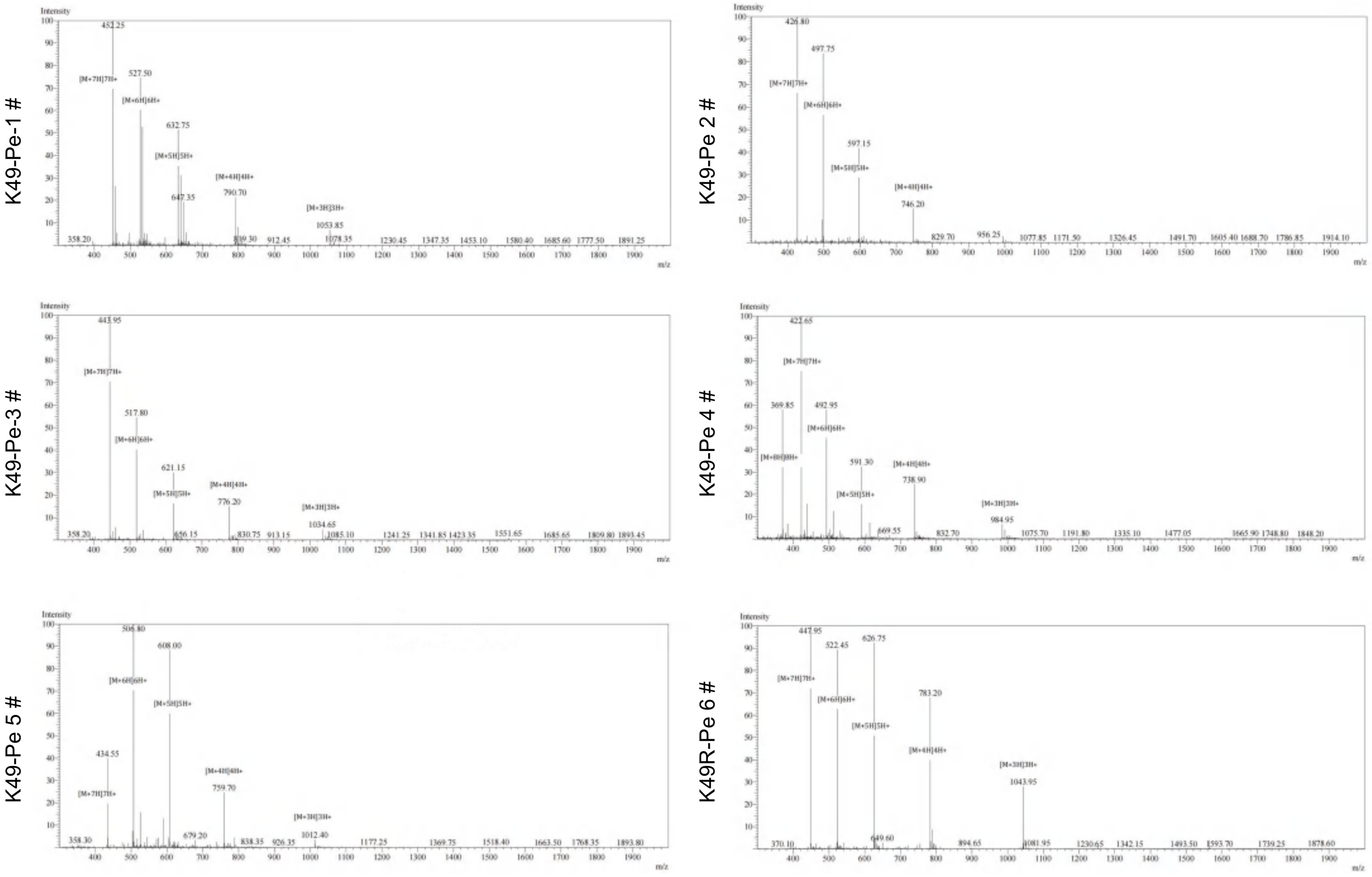

Figure S6. Validation of CRIP1 K49-region peptides

MS spectra of synthetic peptides (CRIP1 K49-Peptide 1–5 and K49R-Peptide 6).

Supplementary Figure 7

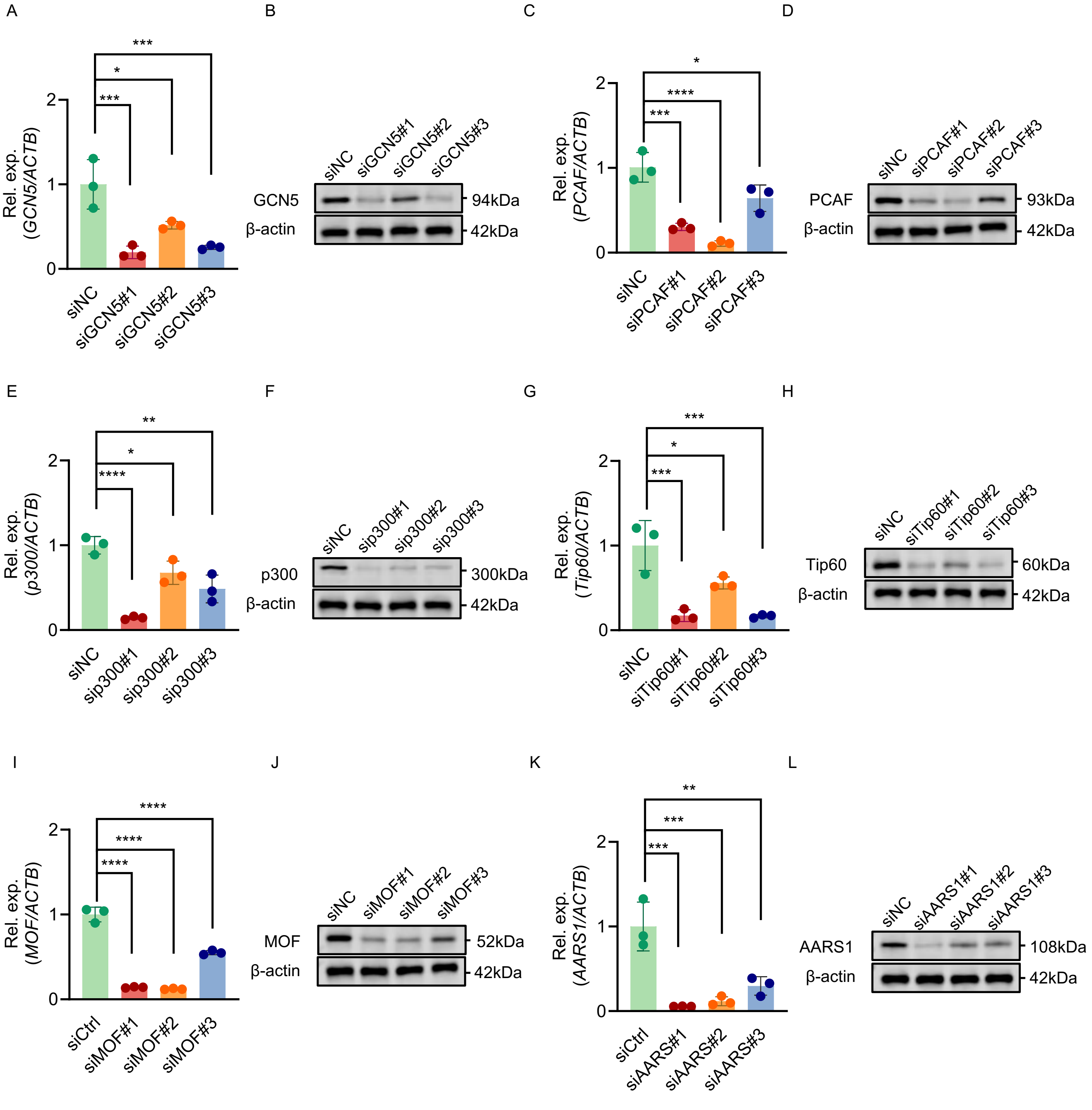

Figure S7. Efficiency validation of lactylation enzyme knockdown

qRT-PCR to quantify mRNA levels and western blotting to evaluate protein expression in RA-FLSs transfected with siCtrl or siRNA targeting *GCN5, PCAF, p300, KAT5, KAT8,* and *AARS1*. **p* < 0.05, ***p* < 0.01, ****p* < 0.001, *****p* < 0.0001. Data are presented as mean ± SEM, and *p*-values are calculated using one-way ANOVA followed by Tukey’s post-hoc test (A, C, E, G, I, K).

Supplementary Figure 8

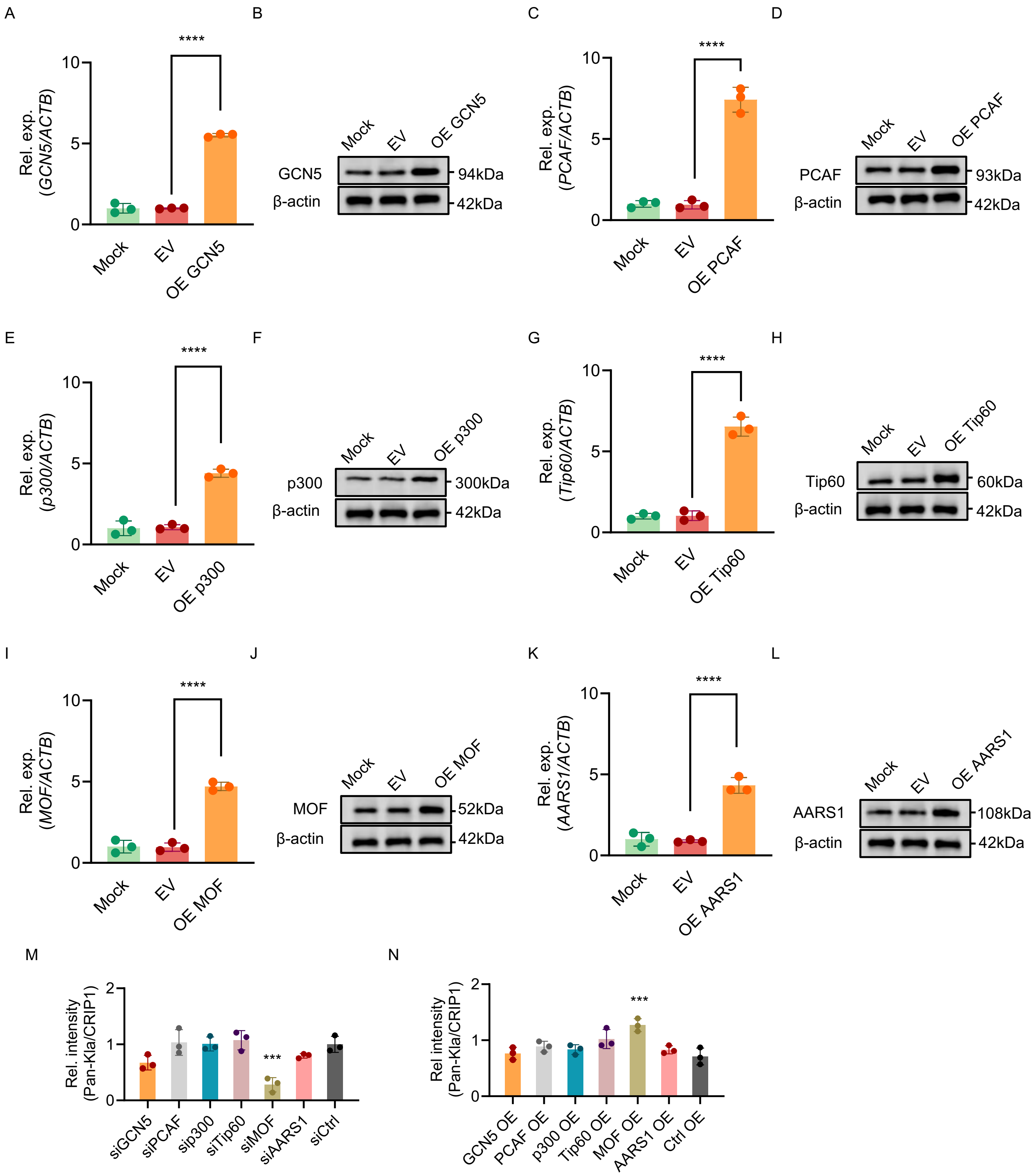

Figure S8. Overexpression of lactylation enzyme

(A–L) qRT-PCR to quantify mRNA levels, and western blotting to evaluate protein expression in RA-FLSs transfected with mock, empty vector, or overexpression constructs (*GCN5, PCAF, p300, Tip60, MOF, and AARS1*). **(M–N)** Quantification analysis of Figure 5A–B. ***$p < 0.001$, ****$p < 0.0001$. Data are presented as mean ± SEM, and *p*-values are calculated using one-way ANOVA followed by Tukey's post-hoc test (A, C, E, G, I, K, M, N).

Supplementary Figure 9

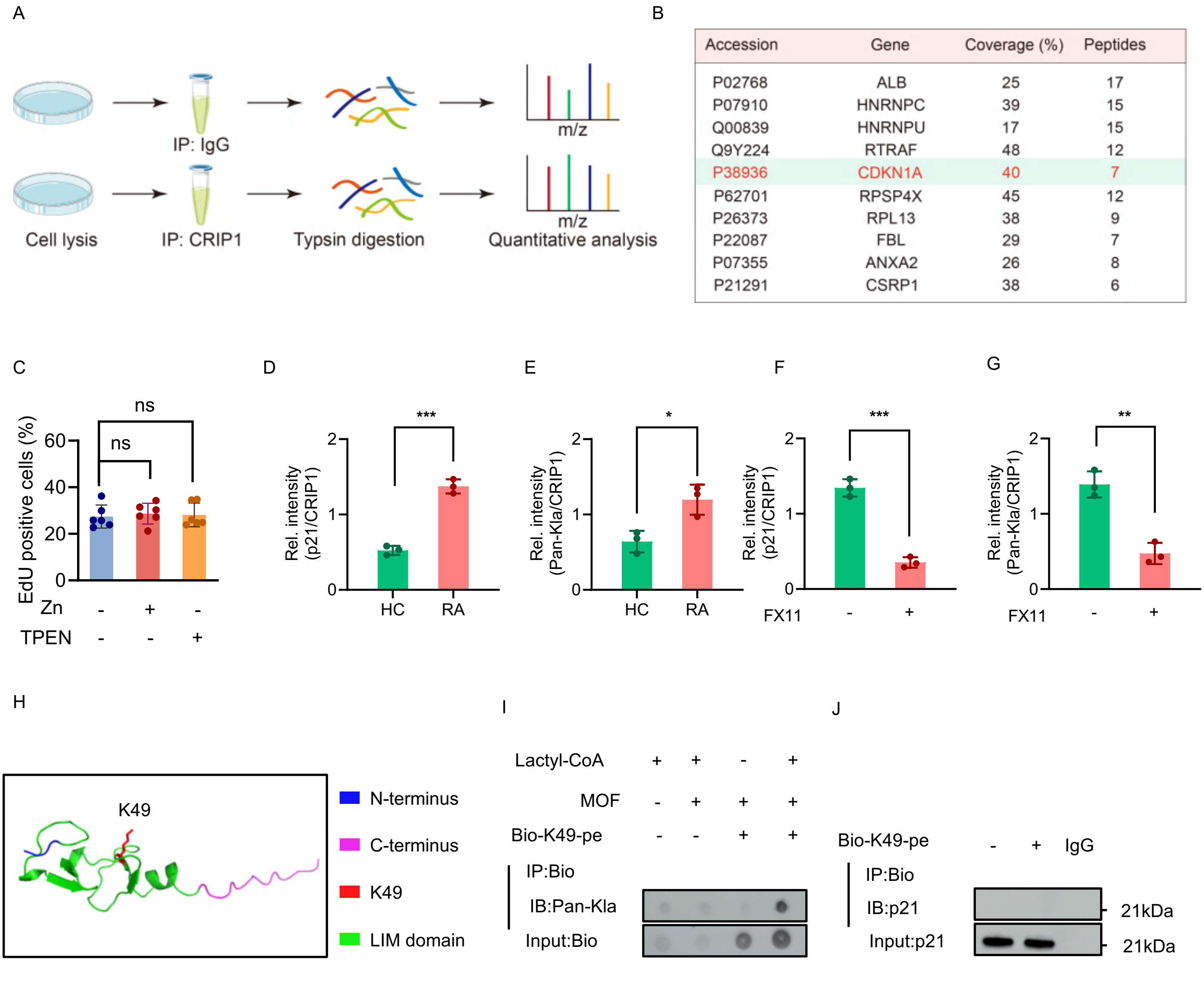

Accession	Gene	Coverage (%)	Peptides
P02768	ALB	25	17
P07910	HNRNPC	39	15
Q00839	HNRNPU	17	15
Q9Y224	RTRAF	48	12
P38936	CDKN1A	40	7
P62701	RPSP4X	45	12
P26373	RPL13	38	9
P22087	FBL	29	7
P07355	ANXA2	26	8
P21291	CSRP1	38	6

Figure S9. Analysis of CRIP1-interacting proteins

(A) Schematic diagram for the immunoprecipitation–mass spectrometry (IP–MS) analysis. CRIP1 or IgG control was immunoprecipitated from cell lysates, followed by trypsin digestion and LC-MS/MS-based quantitative proteomic analysis. **(B)** List of CRIP1-associated proteins identified by IP–MS analysis.**(C)** EdU to detect cell proliferation. RA-FLSs were treated with Zn (40 μM) or TPEN (5 μM).**(D–E)** Quantification analysis in Figure 6E. **(F–G)** Quantification analysis in Figure 6F. **(H)** Structural representation of CRIP1. **(I)** In vitro lactylation assay to assess the lactylation of Bio-tagged peptide. **(J)** Co-IP to assess the interation between Bio-tagged peptide and p21. ns = no significance, *$p < 0.05$, **$p < 0.01$, ***$p < 0.001$. Data are presented as mean ± SEM, and *p*-values are calculated using one-way ANOVA followed by Tukey's post-hoc test (C), or unpaired two-tailed t-test (D, E, F, G).

Supplementary Figure 10

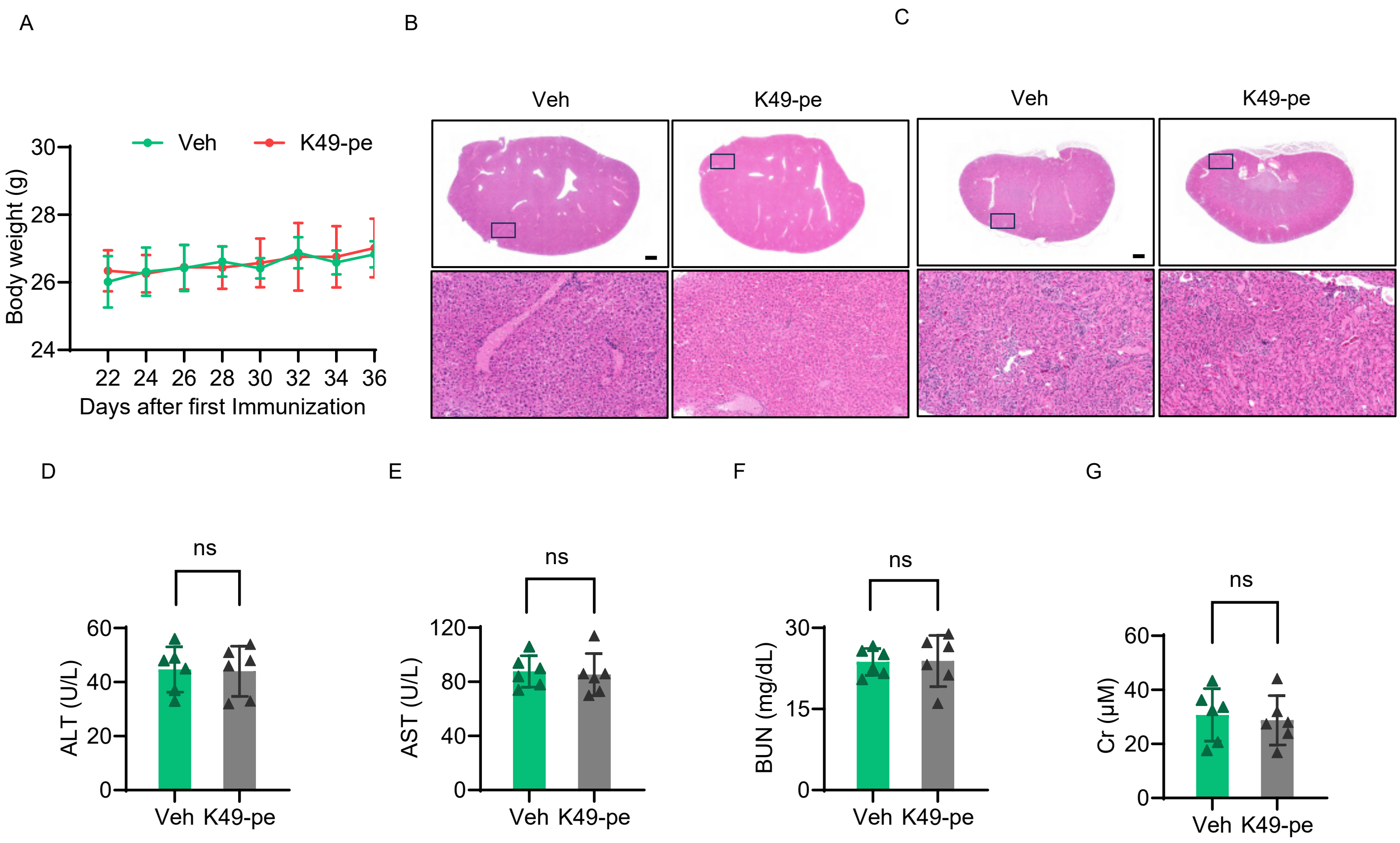

Figure S10. Evaluation of the hepatic and renal toxicity of K49-peptide

(A) Body weight in DBA/1 mice treated with either vehicle or K49-peptide. **(B–C)** Histological analysis of liver and kidney tissues. Scale bar, 500 μm. **(D–G)** Serum ALT, AST, BUN, and Cr levels. ns = no significance. Data are presented as mean ± SEM, and *p*-values are calculated using two-way ANOVA with Bonferroni's post hoc test (A), or unpaired two-tailed t-test (D–G). ALT, alanine transaminase; AST, aspartate aminotransferase; BUN, blood urea nitrogen; Cr, creatinine.

SUPPLEMENTARY TABLE

Supplementary Table 1. Baseline data for patients

	**HC (n=32)**	**RA (n=38)**	***p* value**
Female: Male	20:12	30:10	-
Age (years)	40.16 (19-72)	47.11 (22-75)	0.0546
CRP (IU/ml)	2.65 (0.5-9.1)	30.26 (4-120.5)	<0.0001
ESR (mm/h)	11.72 (5-34)	39.11 (10-96)	<0.0001
Disease duration (years)	-	7.48 (1.1-15.3)	-
DAS28-ESR	-	5.02 (1.9-8.4)	-
Anti-CCP antibody (U/ml)	-	153.44 (19.6-410)	-
RF positive	-	63.2%	-
ANA-positive	-	52.6%	-

Statistical significance was determined using unpaired two-tailed Student's t test. ESR, Erythrocyte sedimentation rate; CRP, C-reactive protein; DAS 28: Disease activity score 28; Anti-CCP antibody: Anti-cyclic citrullinated peptide Antibody; RF: Rheumatoid factor. ANA, Anti-nuclear antibodies.

Supplementary Table 2. Sequence of human oligonucleotides

COL1A1-siRNA #1	sense	CAUUGGUAAUGUUGGUGCUTT
	antisense	AGCACCAACAUUACCAAUGTT
COL1A1-siRNA #2	sense	CCUCAAGAUGUGCCACUCUTT
	antisense	AGAGUGGCACAUCUUGAGGTT
COL1A1-siRNA #3	sense	UCACCCACCGACCAAGAAATT
	antisense	UUUCUUGGUCGGUGGGUGATT
CILP- siRNA #1	sense	GGCUUCUUGUCCAACCCUATT
	antisense	UAGGGUUGGACAAGAAGCCTT
CILP- siRNA #2	sense	GAACUUCAUCAAUGACGAATT
	antisense	UUCGUCAUUGAUGAAGUUCTT
CILP- siRNA #3	sense	GCAUCCUGAAGAUCACAAATT
	antisense	UUUGUGAUCUUCAGGAUGCTT
CNN3- siRNA #1	sense	GCAUCUUUAUGAUCCCAAATT
	antisense	UUUGGGAUCAUAAAGAUGCTT
CNN3- siRNA #2	sense	GGAACAAAUGGUUCGGAAATT
	antisense	UUUCCGAACCAUUUGUUCCTT
CNN3- siRNA #3	sense	GUACCAGAAGAGACAUCUATT
	antisense	UAGAUGUCUCUUCUGGUACTT

AHNAK- siRNA #1	sense	CCACCAUCUACUUUGACAATT
	antisense	UUGUCAAAGUAGAUGGUGGTT
AHNAK- siRNA #2	sense	UGCAGGAGGUGACGCAGAATT
	antisense	UUCUGCGUCACCUCCUGCATT
AHNAK- siRNA #3	sense	GGAUGUAGAUGUUCCCAAATT
	antisense	UUUGGGAACAUCUACAUCCTT
CRIP1- siRNA #1	sense	CGCUGAGCACGAAGGCAAATT
	antisense	UUUGCCUUCGUGCUCAGCGTT
CRIP1- siRNA #2	sense	AGUGCAACAAGGAGGUGUATT
	antisense	UACACCUCCUUGUUGCACUTT
CRIP1- siRNA #3	sense	CUGCCUGAAGUGCGAGAAATT
	antisense	UUUCUCGCACUUCAGGCAGTT
GCN5-siRNA #1	sense	GGCUACCUACAAGGUCAAUUATT
	antisense	UAAUUGACCUUGUAGGUAGCCTT
GCN5-siRNA #2	sense	AGACACCAAGCAGGUCUAUUUTT
	antisense	AAAUAGACCUGCUUGGUGUCUTT
GCN5-siRNA #3	sense	CCACCUGAAGGAGUAUCACAUTT
	antisense	AUGUGAUACUCCUUCAGGUGGTT
PCAF-siRNA #1	sense	CCUAAACCGCAUCAACUAUUGTT
	antisense	CAAUAGUUGAUGCGGUUUAGGTT

PCAF-siRNA #2	sense	GCAGAUACCAAACAAGUUUAUTT
	antisense	AUAAACUUGUUUGGUAUCUGCTT
PCAF-siRNA #3	sense	CGAACUCUAAUCCUCACUCAUTT
	antisense	AUGAGUGAGGAUUAGAGUUCGTT
P300-siRNA #1	sense	CCCGGUGAACUCUCCUAUAAUTT
	antisense	AUUAUAGGAGAGUUCACCGGGTT
P300-siRNA #2	sense	ACCUCGUGAUGCCACUUAUUATT
	antisense	UAAUAAGUGGCAUCACGAGGUTT
P300-siRNA #3	sense	CCAGCCUCAAACUACAAUAAATT
	antisense	UUUAUUGUAGUUUGAGGCUGGTT
Tip60-siRNA #1	sense<br>antisense	CAAGUGUCUUCAGCGUCAUUUTT<br>AAAUGACGCUGAAGACACUUGTT
Tip60-siRNA #2	sense	UCGAAUUGUUUGGGCACUGAUTT
	antisense	AUCAGUGCCCAAACAAUUCGATT
Tip60-siRNA #3	sense	GACUUCAACAAACGUCUGGAUTT
	antisense	AUCCAGACGUUUGUUGAAGUCTT
MOF-siRNA #1	sense	ACAUCGGGAACUACGAAAUUGTT
	antisense	CAAUUUCGUAGUUCCCGAUGUTT
MOF-siRNA #2	sense	AUGGCAAAGACCAUAAGAUUUTT
	antisense	AAAUCUUAUGGUCUUUGCCAUTT
MOF-siRNA #3	sense	GCAAGAUCACUCGCAACCAAATT
	antisense	UUUGGUUGCGAGUGAUCUUGCTT

AARS1-siRNA #1	sense	GUCAACCAGGACGACCCUAAUTT
	antisense	AUUAGGGUCGUCCUGGUUGACTT
AARS1-siRNA #2	sense	ACAUGGUGAAGGACAUCAUUATT
	antisense	UAAUGAUGUCCUUCACCAUGUTT
AARS1-siRNA #3	sense	CCCAGGCAACAUGAAGGAUAATT
	antisense	UUAUCCUUCAUGUUGCCUGGGTT

Supplementary Table 3. Sequence of Primers

ACTB	Forward	CGCGAGAAGATGACCCAGAT
	Reverse	TCACCGGAGTCCATCACGAT
COL1A1	Forward	GAGGGCCAAGACGAAGACATC
	Reverse	CAGATCACGTCATCGCACAAC
CILP	Forward	GCCCTGGTGAGTGGACAAC
	Reverse	GTCAGTGGTCCGAGCCTCTA
CNN3	Forward	GAAGAAGGTCAACGAGTCCTCA
	Reverse	AGTCTGAACCTGGGTCATGTT
AHNAK	Forward	TACCCTTCCTAAGGCTGACATT
	Reverse	TTGGACCCTTGAGTTTTGCAT
CRIP1	Forward	CCTGCCTGAAGTGCGAGAAAT
	Reverse	CCTTTAGGCCCAAACATGGC
PCAF	Forward	GAATCGCCGTGAAGAAAGCG
	Reverse	TACAAGACTCCTCGGCCTTG
P300	Forward	ACCAGGAATGACTTCTAGTTTGA
	Reverse	GGGTTTGCCGGGGTACAATA
Tip60	Forward	AACAAACGTCTGGATGAATGGG
	Reverse	AGGAAGTCCGTTCTTAGTGGG
MOF	Forward	GCTGGACGAGTGGGTAGAC
	Reverse	TTTGGTTGCGAGTGATCTTGC
AARS1	Forward	CCGATGTCCAGAAACGAGTGT

	Reverse	ATGACAAGAGGCTGGTTGGG
IL6	Forward	ACTCACCTCTTCAGAACGAATTG
	Reverse	CCATCTTTGGAAGGTTCAGGTTG
TNF	Forward	CCTCTCTCTAATCAGCCCTCTG
	Reverse	GAGGACCTGGGAGTAGATGAG
IL1B	Forward	ATGATGGCTTATTACAGTGGCAA
	Reverse	GTCGGAGATTCGTAGCTGGA
MMP1	Forward	AAAATTACACGCCAGATTTGCC
	Reverse	GGTGTGACATTACTCCAGAGTTG
MMP9	Forward	AGACCTGGGCAGATTCCAAAC
	Reverse	CGGCAAGTCTTCCGAGTAGT
