# Supplementary material for: Lactate programs CRIP1 protein lactylation to drive synovial proliferation in rheumatoid arthritis
Source: JCI Insight. 2026 Jun 8;11(11):e200928. doi: 10.1172/jci.insight.200928 (PMC13313492; doi:10.1172/jci.insight.200928)

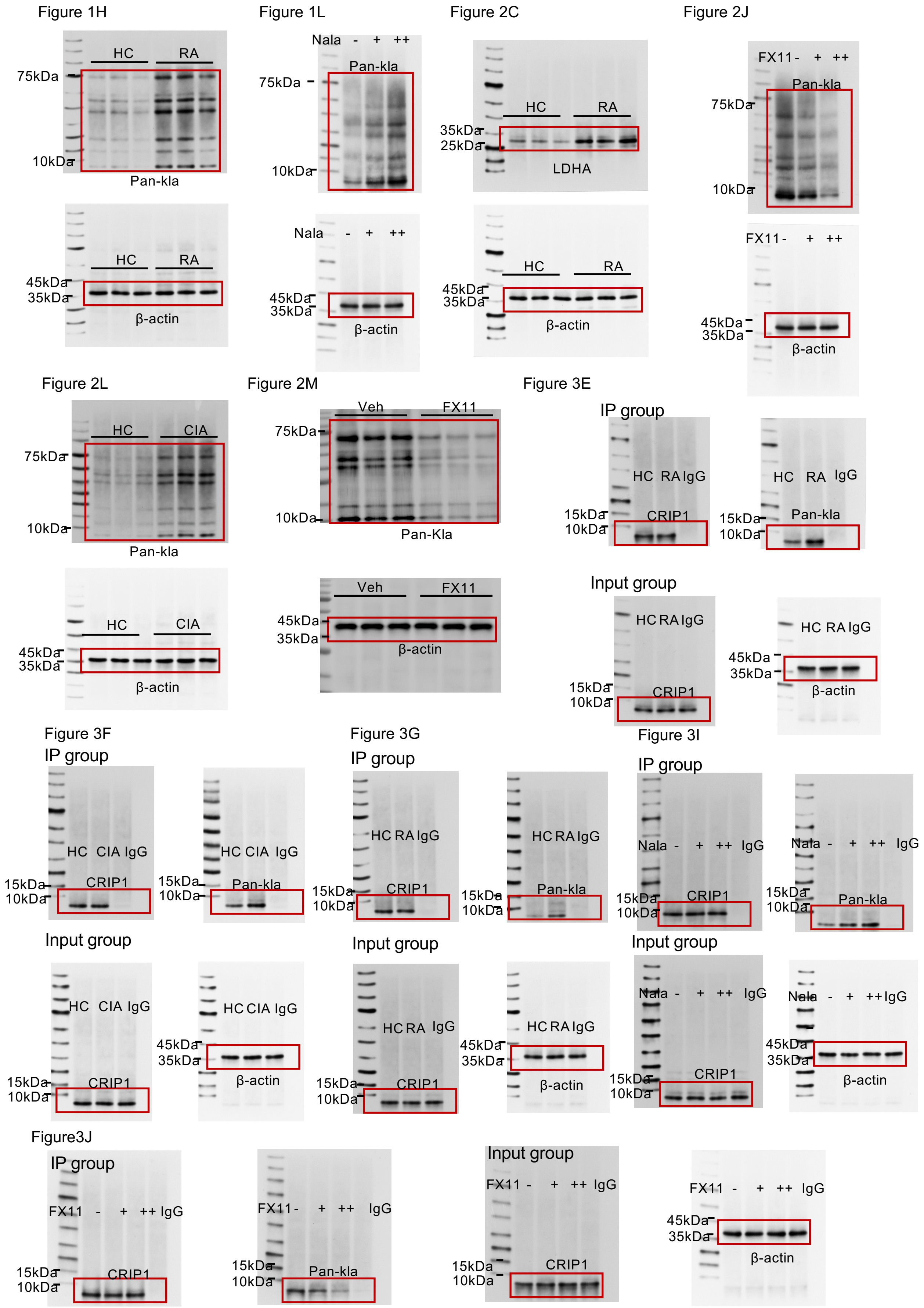
Figure 1H
HC
RA
75kDa
10kDa
Pan-kla
HC
RA
45kDa
35kDa
β-actin
Figure 1L
Nala - + ++
Pan-kla
75kDa
10kDa
Nala - + ++
45kDa
35kDa
β-actin
Figure 2C
HC
RA
35kDa
25kDa
LDHA
HC
RA
45kDa
35kDa
β-actin
Figure 2J
FX11 - + ++
Pan-kla
75kDa
10kDa
FX11 - + ++
45kDa
35kDa
β-actin
Figure 2L
HC
CIA
75kDa
10kDa
Pan-kla
HC
CIA
45kDa
35kDa
β-actin
Figure 2M
Veh
FX11
75kDa
10kDa
Pan-Kla
Veh
FX11
45kDa
35kDa
β-actin
Figure 3E
IP group
HC RA IgG
15kDa
10kDa
CRIP1
HC RA IgG
15kDa
10kDa
Pan-kla
Input group
HC RA IgG
15kDa
10kDa
CRIP1
HC RA IgG
45kDa
35kDa
β-actin
Figure 3F
IP group
HC CIA IgG
15kDa
10kDa
CRIP1
HC CIA IgG
15kDa
10kDa
Pan-kla
Input group
HC CIA IgG
15kDa
10kDa
CRIP1
HC CIA IgG
45kDa
35kDa
β-actin
Figure 3G
IP group
HC RA IgG
15kDa
10kDa
CRIP1
HC RA IgG
15kDa
10kDa
Pan-kla
Input group
HC RA IgG
15kDa
10kDa
CRIP1
HC RA IgG
45kDa
35kDa
β-actin
Figure 3I
IP group
Nala - + ++ IgG
15kDa
10kDa
CRIP1
Nala - + ++ IgG
15kDa
10kDa
Pan-kla
Input group
Nala - + ++ IgG
15kDa
10kDa
CRIP1
Nala - + ++ IgG
45kDa
35kDa
β-actin
Figure3J
IP group
FX11 - + ++ IgG
15kDa
10kDa
CRIP1
FX11 - + ++ IgG
15kDa
10kDa
Pan-kla
Input group
FX11 - + ++ IgG
15kDa
10kDa
CRIP1
FX11 - + ++ IgG
45kDa
35kDa
β-actin

Figure 4C

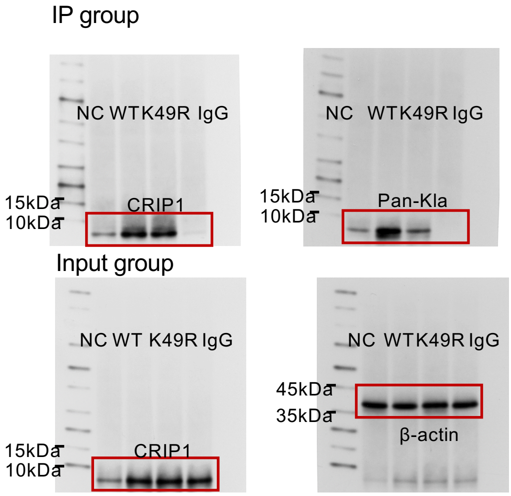

Figure 4H

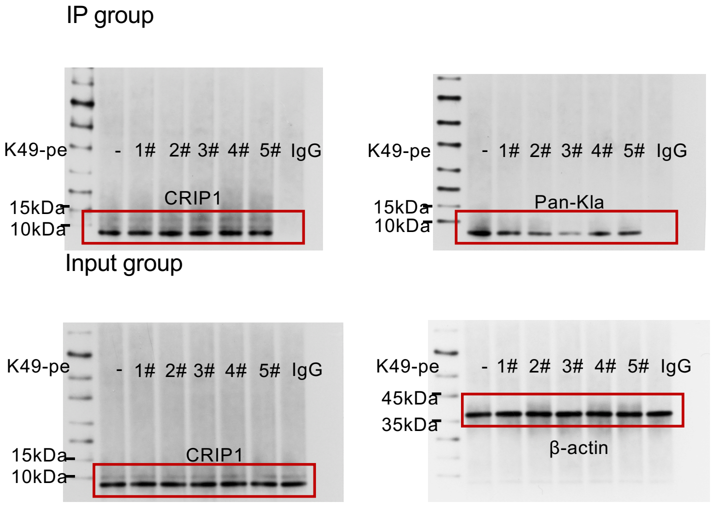

Figure 4I

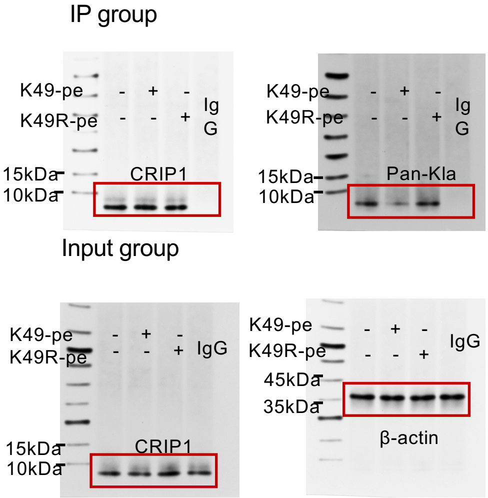

Figure 5A

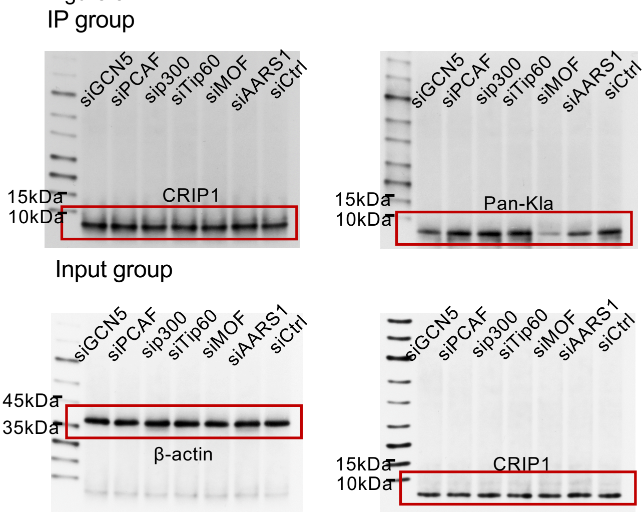

Figure 5B

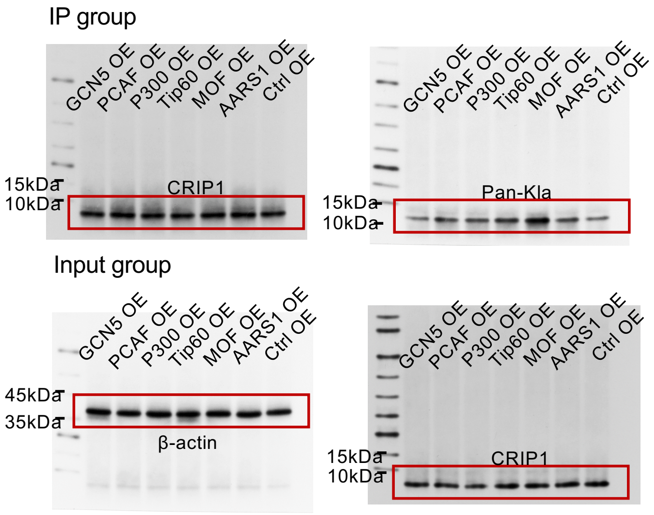

Figure 5C

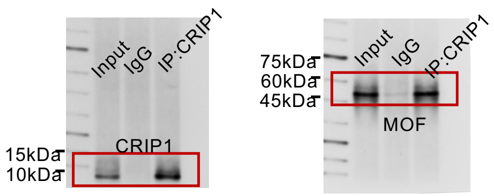

Figure 5D

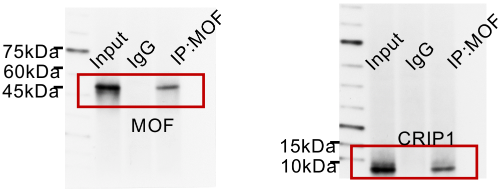

Figure 5G

IP group

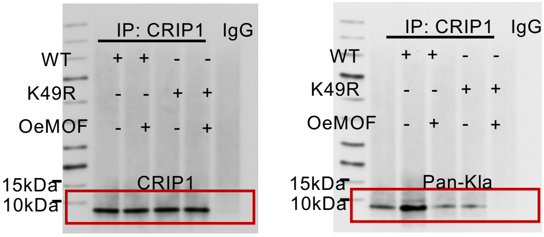

Input group

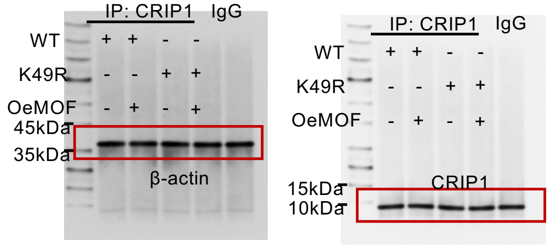

Figure 6E

IP group

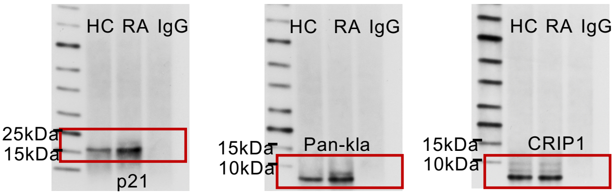

Input group

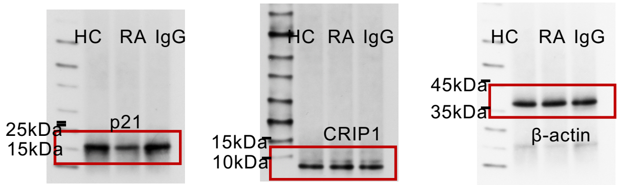

Figure 6F

IP group

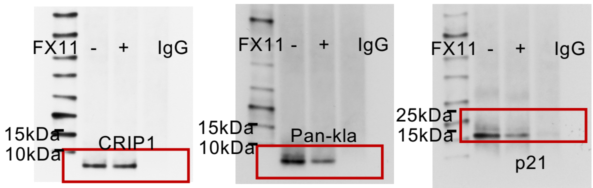

Input group

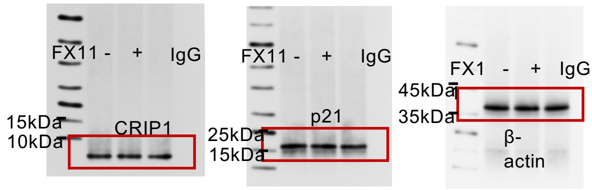

Figure 6L

IP group

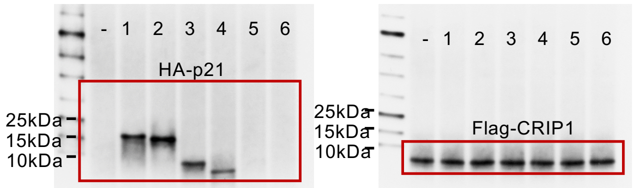

Input group

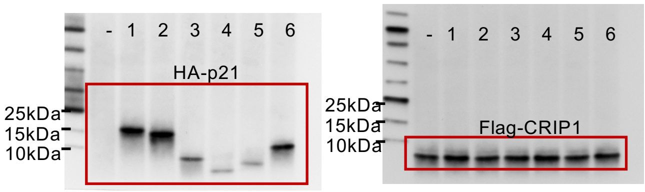

Figure 6J

IP group

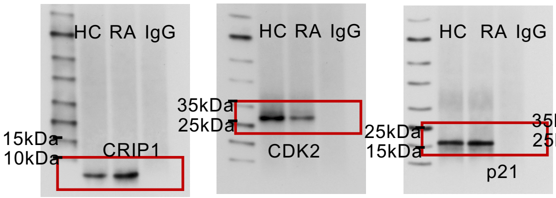

Input group

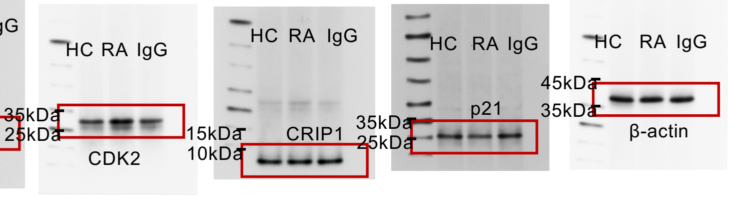

Figure 6K

IP group

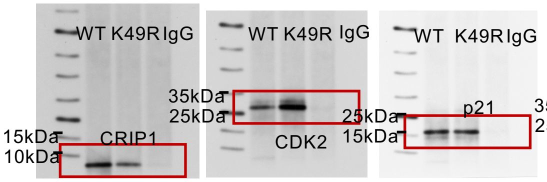

Input group

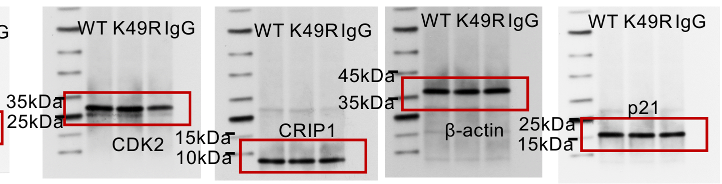

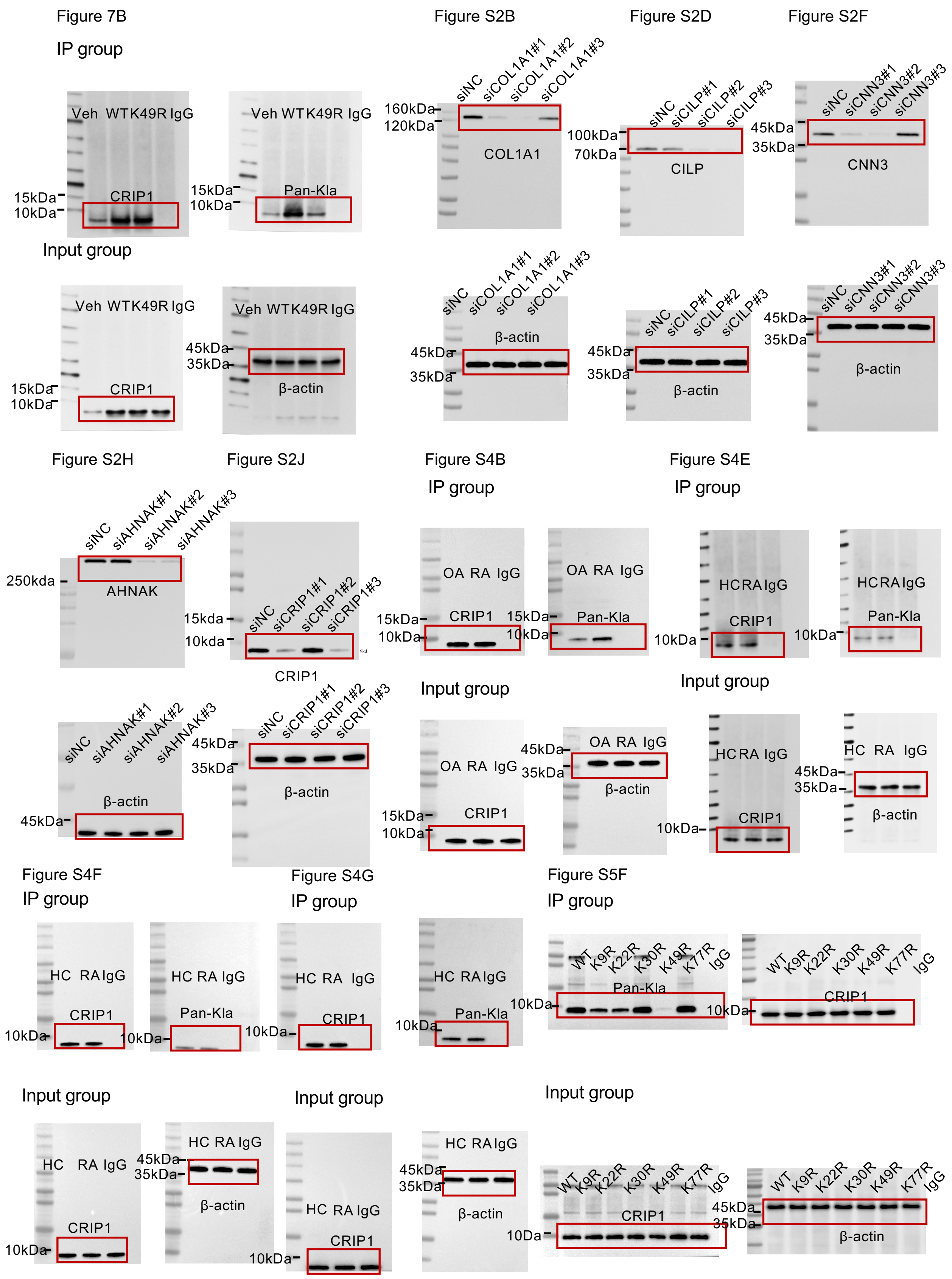
Figure 7B
IP group
Veh WTK49R IgG
15kDa
10kDa
CRIP1
Veh WTK49R IgG
15kDa
10kDa
Pan-Kla
Input group
Veh WTK49R IgG
15kDa
10kDa
CRIP1
Veh WTK49R IgG
45kDa
35kDa
β-actin
Figure S2B
siNC
siCOL1A1#1
siCOL1A1#2
siCOL1A1#3
160kDa
120kDa
COL1A1
siNC
siCOL1A1#1
siCOL1A1#2
siCOL1A1#3
β-actin
45kDa
35kDa
Figure S2D
siNC
siCILP#1
siCILP#2
siCILP#3
100kDa
70kDa
CILP
siNC
siCILP#1
siCILP#2
siCILP#3
45kDa
35kDa
β-actin
Figure S2F
siNC
siCNN3#1
siCNN3#2
siCNN3#3
45kDa
35kDa
CNN3
siNC
siCNN3#1
siCNN3#2
siCNN3#3
45kDa
35kDa
β-actin
Figure S2H
siNC
siAHNAK#1
siAHNAK#2
siAHNAK#3
250kda
AHNAK
siNC
siAHNAK#1
siAHNAK#2
siAHNAK#3
β-actin
45kDa
Figure S2J
15kda
10kda
siNC
siCRIP1#1
siCRIP1#2
siCRIP1#3
CRIP1
siNC
siCRIP1#1
siCRIP1#2
siCRIP1#3
45kDa
35kDa
β-actin
Figure S4B
IP group
OA RA IgG
15kDa
10kDa
CRIP1
OA RA IgG
15kDa
10kDa
Pan-Kla
Input group
OA RA IgG
15kDa
10kDa
CRIP1
OA RA IgG
45kDa
35kDa
β-actin
Figure S4E
IP group
HCRAIgG
10kDa
CRIP1
HCRAIgG
10kDa
Pan-Kla
Input group
HCRA IgG
10kDa
CRIP1
HC RA IgG
45kDa
35kDa
β-actin
Figure S4F
IP group
HC RAIgG
CRIP1
10kDa
HC RA IgG
Pan-Kla
10kDa
Input group
HC RA IgG
CRIP1
10kDa
HC RA IgG
45kDa
35kDa
β-actin
Figure S4G
IP group
HC RA IgG
CRIP1
10kDa
HC RAIgG
Pan-Kla
10kDa
Input group
HC RA IgG
CRIP1
10kDa
HC RAIgG
45kDa
35kDa
β-actin
Figure S5F
IP group
WT K9R K22R K30R K49R K77R IgG
Pan-Kla
10kDa
WT K9R K22R K30R K49R K77R IgG
CRIP1
10kDa
Input group
WT K9R K22R K30R K49R K77R IgG
CRIP1
10Da
WT K9R K22R K30R K49R K77R IgG
45kDa
35kDa
β-actin

Figure S7B Figure S8B Figure S7D Figure S8D Figure S7F Figure S8F

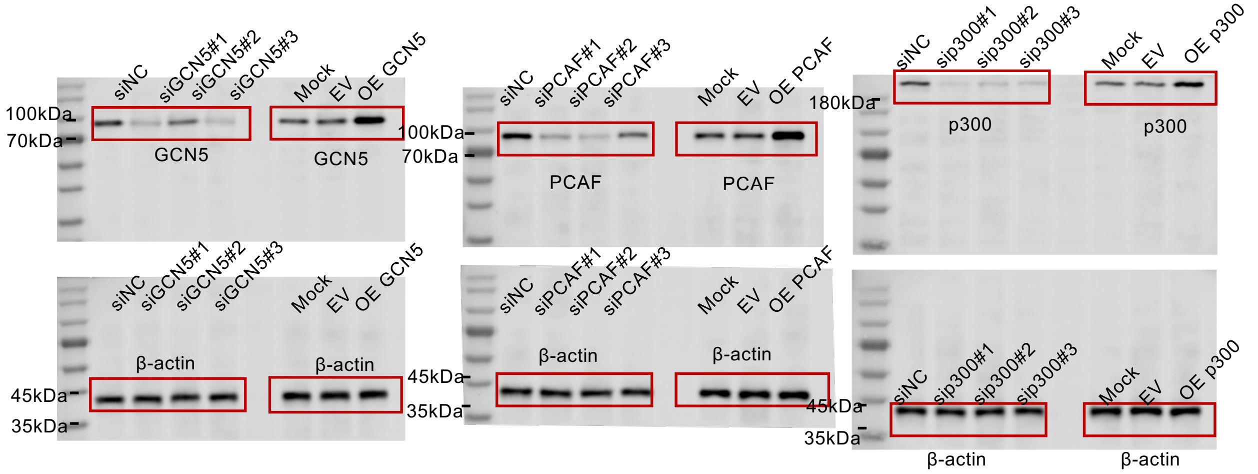

Figure S7H Figure S8H Figure S7J Figure S8J Figure S8L Figure S8L

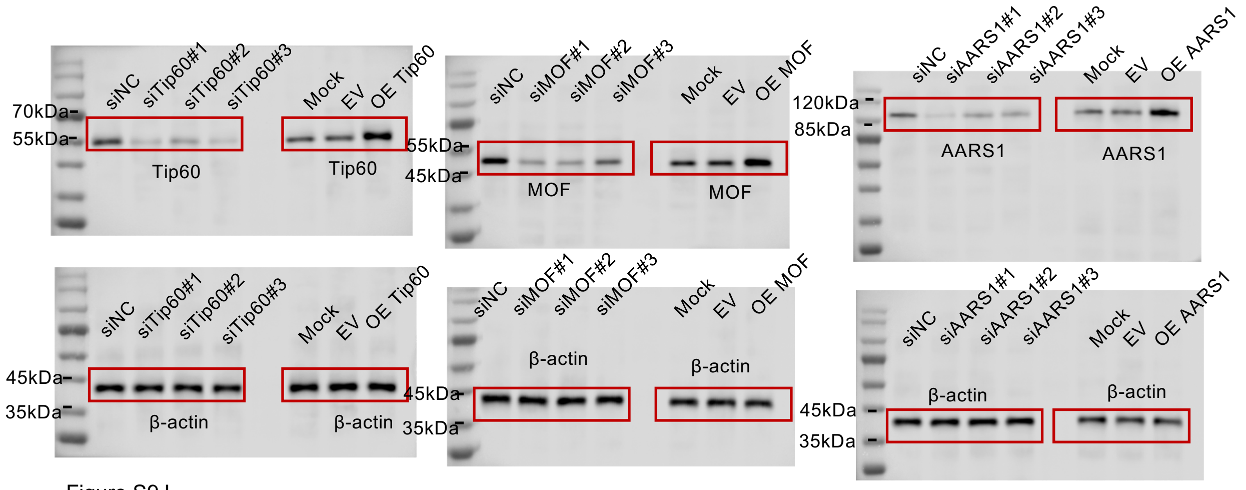

Figure S9J

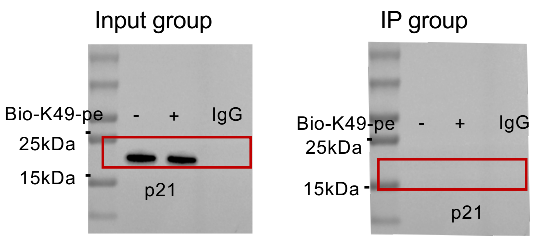

Supplement: Unedited blot and gel images [file jciinsight-11-200928-s035.pdf]
